# Temporal changes in glucose metabolism reflect polarization in resident and monocyte-derived macrophages after myocardial infarction

**DOI:** 10.3389/fcvm.2023.1136252

**Published:** 2023-05-05

**Authors:** Alan J. Mouton, Nikaela M. Aitken, Sydney P. Moak, Jussara M. do Carmo, Alexandre A. da Silva, Ana C. M. Omoto, Xuan Li, Zhen Wang, Alexandra C. Schrimpe-Rutledge, Simona G. Codreanu, Stacy D. Sherrod, John A. McLean, John E. Hall

**Affiliations:** ^1^ Department of Physiology and Biophysics, University of Mississippi Medical Center, Jackson, United States; ^2^ Mississippi Center for Obesity Research, University of Mississippi Medical Center, Jackson, United States; ^3^ Department of Chemistry and Center for Innovative Technology, Vanderbilt University, Nashville, TN, United States

**Keywords:** glycolysis, immunometabolism, macrophage, inflammation, heart failure

## Abstract

**Introduction:**

Metabolic reprogramming from glycolysis to the mitochondrial tricarboxylic acid (TCA) cycle and oxidative phosphorylation may mediate macrophage polarization from the pro-inflammatory M1 to the anti-inflammatory M2 phenotype. We hypothesized that changes in cardiac macrophage glucose metabolism would reflect polarization status after myocardial infarction (MI), ranging from the early inflammatory phase to the later wound healing phase.

**Methods:**

MI was induced by permanent ligation of the left coronary artery in adult male C57BL/6J mice for 1 (D1), 3 (D3), or 7 (D7) days. Infarct macrophages were subjected to metabolic flux analysis or gene expression analysis. Monocyte versus resident cardiac macrophage metabolism was assessed using mice lacking the Ccr2 gene (CCR2 KO).

**Results:**

By flow cytometry and RT-PCR, D1 macrophages exhibited an M1 phenotype while D7 macrophages exhibited an M2 phenotype. Macrophage glycolysis (extracellular acidification rate) was increased at D1 and D3, returning to basal levels at D7. Glucose oxidation (oxygen consumption rate) was decreased at D3, returning to basal levels at D7. At D1, glycolytic genes were elevated (Gapdh, Ldha, Pkm2), while TCA cycle genes were elevated at D3 (Idh1 and Idh2) and D7 (Pdha1, Idh1/2, Sdha/b). Surprisingly, Slc2a1 and Hk1/2 were increased at D7, as well as pentose phosphate pathway (PPP) genes (G6pdx, G6pd2, Pgd, Rpia, Taldo1), indicating increased PPP activity. Macrophages from CCR2 KO mice showed decreased glycolysis and increased glucose oxidation at D3, and decreases in Ldha and Pkm2 expression. Administration of dichloroacetate, a pyruvate dehydrogenase kinase inhibitor, robustly decreased pyruvate dehydrogenase phosphorylation in the non-infarcted remote zone, but did not affect macrophage phenotype or metabolism in the infarct zone.

**Discussion:**

Our results indicate that changes in glucose metabolism and the PPP underlie macrophage polarization following MI, and that metabolic reprogramming is a key feature of monocyte-derived but not resident macrophages.

## 1. Introduction

Almost 1 million adults in the United States suffer a myocardial infarction (MI) every year (1). In surviving patients, MI often progresses to heart failure (2). The pathophysiology of MI is characterized by transient or permanent ischemia to a portion of the left ventricle (LV), which undergoes necrotic myocyte death followed by a robust inflammatory response. Inflammation orchestrates tissue healing and remodeling to form a permanent scar (2–5). Macrophages play a critical role in all phases of healing, including initiating and propagating the early inflammatory response, while later reverting towards a reparative phenotype to promote myofibroblast activation, collagen deposition, and timely resolution of inflammation (2, 6). After MI, resident cardiac macrophages undergo cell death in the infarct area and are replaced by monocytes, which use C-C motif chemokine receptor type 2 (CCR2) to migrate to and infiltrate the infarcted area and are more inflammatory than resident macrophages (3, 6–10).

Understanding the mechanisms of macrophage polarization has been a critical focus point for developing therapies to target inflammation during MI and heart failure (2, 6). An important mechanism by which macrophages polarize towards pro- (M1) and anti-inflammatory (M2) phenotypes is *via* changes in metabolism, or metabolic reprogramming (11–14). Metabolic reprogramming in M1-polarized macrophages is characterized by an increase in glycolytic metabolism and a decrease in mitochondrial oxidative phosphorylation (OXPHOS), as well as truncation of the tricarboxylic acid (TCA) cycle and accumulation of pro-inflammatory intermediates such as succinate (14). Another key feature of the M1 phenotype is upregulation of the pentose phosphate pathway (PPP), which generates NADPH required for synthesis of pro-inflammatory lipid mediators (13, 14). Glycolytic reprogramming is mediated by hypoxia-inducible factor 1-alpha (HIF-1α), which responds to hypoxia as well as inflammatory signals to activate a glycolytic gene program (3, 12, 15). On the other hand, M2 macrophages possess a fully intact TCA cycle and rely on OXPHOS, and downregulate glycolytic metabolism (14).

We previously showed by transcriptomics that glycolytic genes are heavily induced early after MI, while mitochondrial gene networks are induced at later time points (3). We have also shown that administration of dimethyl fumarate (DMF), an immunometabolic and anti-inflammatory agent used for psoriasis and multiple sclerosis, improves post-MI outcomes in association with attenuated macrophage metabolic reprogramming (16). In this study we hypothesized that macrophage glycolytic metabolic reprogramming occurs in a time-dependent manner reflective of polarization status. We also hypothesized that resident cardiac macrophages, which are less inflammatory than infiltrating monocytes, are also less glycolytic. We found that macrophages exhibit glycolytic reprogramming up to 3 days after MI, which is reversed by day 7. We also show that PPP genes are increased at day 7, indicating a potential novel role for the PPP in reparative macrophages during MI. We also demonstrate that resident cardiac CCR2- macrophages do not undergo glycolytic metabolic reprogramming after MI, indicating that glycolytic metabolic reprogramming may be a key feature of infiltrating monocytes. Thus, glucose metabolism may be a potential therapeutic strategy for targeting monocyte-mediated inflammation early after MI.

## 2. Materials and methods

### 2.1. Animal groups and numbers

Experiments were conducted in accordance with Animals in Research: Reporting *in vivo* Experiments (ARRIVE) guidelines (17). For comparisons between days 1, 3, and 7, 172 mice were used, with 88 mice surviving (Supplementary Figure S1). Of the mice that died, 2 died during the surgery and were excluded from the survival analysis. 4 mice did not develop an infarction (>30% ejection fraction at day 1 echo) and were euthanized. For day 1, 54 mice were pooled to obtain macrophages for downstream analysis (Seahorse and real-time PCR) as described previously (3); 15 mice were pooled for day 3, and 19 were pooled for day 7. For the CCR2 KO studies, 8 mice were used for day 1 (*n* = 4 each group). For cardiac macrophage extraction, 29 mice were pooled to obtain macrophages for downstream analysis. For studies involving DCA, 27 total mice were used (*n* = 13 vehicle, *n* = 14 DCA), which were pooled for cardiac macrophage isolation.

### 2.2. Permanent ligation model of MI

All protocols were approved by the University of Mississippi Medical Center Institutional Animal Care and Use Committee. Adult (15–20 weeks old) male C57BL/6J or CCR2 KO mice (strain #004999; Jackson Labs; Bar Harbor, ME) were used in this study. To induce MI, a non-reperfused permanent ligation of the left coronary artery was performed. Mice were anesthetized (2% isoflurane), immobilized in the supine position on a heating pad (37°C), intubated and connected to a mini-ventilator (Harvard Apparatus; 250–300 µl stroke volume, 250–300 breaths/min). An incision was made on the left side of the chest cavity to expose the ribs, and the heart was exposed between the 3rd and 4th ribs. The pericardium was removed and the left coronary artery was ligated just below the left atrium using 8-0 suture. Ischemia was confirmed by blanching of the LV below the suture. Dichloroacetate (DCA) was administered by intraperitoneal injection (100 mg/kg) 2 h after MI and twice daily on days 1 and 2 after MI. Control mice received an equal volume of vehicle (saline). DCA was also supplemented in the drinking water (110 mM) (18).

### 2.3. Echocardiography

Cardiac function was assessed by echocardiography (VEVO 3,100, VisualSonics). Mice were anesthetized (2% isoflurane) and laid supine on a heating surface (37°C). B-mode images were obtained along the long and short-axis, and M-mode images obtained in the short-axis, and processed using VEVOLAB software (Version 3.2.6). Ejection fraction (EF) and LV systolic and diastolic volumes were calculated from long-axis B-mode images, while wall thickness and LV dimensions were obtained from M-mode short-axis images.

### 2.4. Flow cytometry

Cardiac immune cells were quantified using flow cytometry in collaboration with the UMMC Flow Cytometry Core. Hearts were minced and digested using a solution of type II collagenase (600 U/ml, Worthington) and 60 U/ml DNase I (Sigma-Aldrich) for 1 h at 37°C. The digested suspension was then filtered (30 µm) to generate a single-cell suspension. Non-specific Fc-receptor binding sites were blocked with anti-CD16/32 (BioLegend). Cells were then stained with the following fluorophore-conjugated antibodies and concentrations: CD45-APC (BD Pharmingen #559864), 1:400; CD11b-PE-Cy7 (BD Pharmingen #552850), 1:400; Ly6G-PE (BD Pharmingen #551461), 1:400; Ly6C-FITC BD (Pharmingen #553126), 1:100. Dead cells were labeled with propidium iodide and excluded from analysis. The single-cell suspensions were then sorted using a flow cytometer (LSR II, BD Biosciences) at a rate event of <400 events/second and the data were analyzed using FCS Express software (De Novo Software).

### 2.5. Macrophage Isolation

Highly pure macrophage populations were isolated from the infarcted area of the heart as previously described (3, 16). Hearts were digested as described above. Macrophages were then selected by magnetic microbead separation by first removing Ly6G neutrophils (Ly6G microbeads, Miltenyi #130-120-337) and collecting the remaining CD11b + cells (CD11b microbeads, Miltenyi #130-126-725). Macrophages were then plated in 24-well Seahorse cultureware in RPMI + 0.1% FBS for 2 h for Seahorse experiments or in 6-well plates for RNA extraction and real-time PCR. The 2 h incubation period was previously established to obtain optimal RNA quality while maintaining the *in vivo* phenotype, as assessed previously (3). For some experiments, macrophages were pooled from different mice to generate enough cells for downstream assays.

### 2.6. Seahorse extracellular flux analysis

Macrophage metabolism was assessed by extracellular flux analysis (Seahorse XFe24, Agilent). To assess glycolysis and glucose oxidation, the glycolysis stress test was performed (Agilent #103020-100). Macrophages were plated at a density of 5 × 10^5^ cells per well and nutrient starved for 1 h in basal RPMI media in a CO_2_-free incubator (37°C). The plate was then inserted into the machine, and following basal measurements, serial injections of glucose (10 mM), oligomycin (1 µM), and 2-deoxyglucose (50 mM) were performed. For each injection, 3 separate measurements of extracellular acidification rate (ECAR) and oxygen consumption rate (OCR) were performed over 3 time intervals. Basal glycolysis was assessed as ECAR_glucose_–ECAR_basal_, while maximal glycolysis was ECAR_oligomycin_–ECAR_glucose_. Glucose oxidation was assessed as OCR_glucose_–OCR_basal_.

### 2.7. Real time polymerase chain reaction

Real-time polymerase chain reaction (RT-PCR) was used to assess changes in macrophage gene expression. RNA was extracted by the UMMC Molecular Genomics Core (PureLink RNA Mini-Kit; Fisher). RNA was reverse-transcribed into cDNA (iScript Reverse Transcription Super Mix; Bio-Rad). The PCR reaction was performed using cDNA (50 ng) mixed with SYBR Green Master Mix (Bio-Rad) and the following primers: *Il1b, Slc2a1, Gapdh, Ldha, Hif1a, Pdk1, Pdha1, Irg1, Sdha, Sdhb, Idh1, Idh2, Pkm2, Pfkfb3, Hk1, Hk2, G6pdx, G6pd2, Pgd, Rpia, Tkt, Taldo1, Il10, Tgfb1,* and *Actb* (Supplementary Table S1)*.* The reaction was performed over 40 cycles in a thermocycler (StepOnePlus™ Real-Time PCR System; Applied Biosystems). Changes in gene expression were calculated using the ΔΔCt method using *Actb* as a housekeeping gene.

### 2.8. Immunoblotting

Levels of pyruvate dehydrogenase (PDH) phosphorylation in the remote area of the LV were assessed by immunoblotting. LV tissue was homogenized in T-PER buffer in the presence of protease and phosphatase inhibitors. Protein concentration was assessed by BCA assay. Protein (20 µg) was separated by sodium dodecyl sulfide-polyacrylamide gel electrophoresis (SDS-PAGE) and transferred to nitrocellulose membranes. The membrane was blocked for 1 h, stained for total protein (Revert™ 700 Total Protein Stain Kit; Li-Cor #P/N 926-11010), and incubated with primary antibodies for phospho-PDH (Serine 293; 1:1,000; Cell Signaling Technologies #31866) or PDH (1:1,000; Cell Signaling #2784) overnight at 4°C. Membranes were then incubated in a secondary antibody IRDye® 8000CW Donkey Anti-Rabbit IgG; 1:10,000; Li-Cor) for 2 h at room temperature. Blots were imaged using the Odyssey® CLx Imaging System. Protein expression was normalized to total protein.

### 2.9. Immunofluorescence and infarct size measurement

Macrophage subtypes were further evaluated by immunofluorescent staining of LV tissue using the Opal Manual IHC Kit (Perkin Elmer; Boston, MA) as described previously (5, 16). LV tissue was fixed and embedded in paraffin, then sectioned at 5 µm. Slides were incubated overnight in primary antibody against the following targets: Mac-2 (1:200 dilution; R&D Systems #AF1197), CD206 (1:200 dilution; Cell Signaling Technology #87887), and CCR2 (1:10 dilution; Abcam #273050). The next day, HRP-conjugated secondary antibody was applied, and conjugated to the following fluorophores: Opal 520 (CCR2), Opal 570 (CD206), or Opal 650 (Mac-2), then counterstained with DAPI. Sections were imaged randomly at 20X using the Mantra System (Perkin Elmer) mounted on a fluorescent microscope. Cell counts were quantified using inForm software (Perkin Elmer). Primary antibody specificity was confirmed by staining with secondary antibody only. Infarct size was assessed by Mac-2 positivity in whole LV sections at 4X magnification using ImageJ software.

### 2.10. Statistics

Data was collected and analyzed using Microsoft Excel 2016 and GraphPad 9 (Prism). Student's *t*-test was used to assess differences between two groups, and 1-way ANOVA followed by Tukey's post-hoc analysis was used to assess differences between time points. Data normality was assessed by the Shapiro–Wilk test in GraphPad. Data are presented as mean ± SEM. A *p*-value of less than 0.05 was considered statistically significant.

## 3. Results

### 3.1. Survival and cardiac function after MI

The 7-day survival rate after MI was 53% (Supplementary Figure S1). The echocardiographic and morphological parameters are displayed in Supplementary Table S2. As expected, there was progressive deterioration of cardiac function after MI (3). EF was significantly decreased at all post-MI days. Infarct wall (LVAWd) thinning was observed at days 1 and 3, which was further decreased at day 7. Thinning of the non-infarcted posterior wall (LVPWd) was also observed at day 7. LV dilation as assessed by increased internal diameter at diastole (LVIDd) and end-diastolic volume (LVEDV) was also observed at days 1 and 3, and further increased at day 7. Increased LV mass was observed at days 3 and 7, and lung weight was increased at day 7, indicating pulmonary congestion (3, 16).

### 3.2. Changes in macrophage subtypes after MI

**Figure 1:**
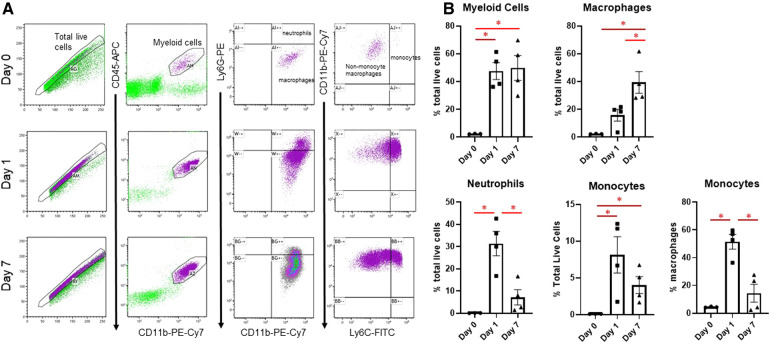
Flow cytometry assessment of cardiac immune cells after MI. (**A**) Representative flow cytometry plots of cardiac immune cells at day 0, day 1, and day 7 after MI. After gating for total live cells, myeloid cells were determined by CD45-APC and CD11b-PE-Cy7 expression. This population was then separated into neutrophils and macrophages based on Ly6G-PE, and Ly6G- macrophages were divided into CD11b + Ly6G-Ly6C^high^ monocytes and CD11b + Ly6G-Ly6C^low^ macrophages. (**B**) Quantification of sorted cells. *p < 0.05, *n* = 3–4 per group.

Changes in infarct immune cell numbers and subtypes after MI were quantified by flow cytometry at days 1 and 7, at which time points the M1 and M2 phenotypes predominate, respectively (3) (Figure 1). Myeloid cells (CD45 + CD11b+) were similarly increased at both days 1 and 7. At day 1, there was an increase in neutrophils (CD45 + CD11b + Ly6G+), as described previously (10), and monocytes (CD45 + CD11b + Ly6C^high^), which returned to basal levels by day 7. At day 7, there were substantially more CD11b + Ly6G-Ly6C^low^ macrophages than day 1. These results indicate that at day 1 after MI, which captures the peak of the inflammatory phase, neutrophils and CD11b + Ly6G-Ly6C^high^ monocytes are predominant in the infarcted LV, while at day 7, anti-inflammatory Ly6C^low^ macrophages predominate.

Macrophage subpopulations in the border, infarct, and remote areas of the heart were further characterized by immunofluorescence. Using Mac-2 (Galectin-3) as a pan-macrophage marker (3), we found that macrophages were increased in the border zone at days 1, 3, and 7 after MI, with no changes in the remote area (Supplementary Figure S2). Confirming our flow cytometry results, we found that macrophage infiltration into the heart began at day 1, and further increased at days 3 and 7. Using CD206 as an M2 macrophage marker (19), we found that M2 macrophages were elevated in the infarct at all days after MI, reaching the highest levels at day 7. Finally, using CCR2 as a monocyte marker (20), we found that monocytes were substantially increased in the infarct at day 1, then progressively decreased by day 7, although still slightly increased. We also found increased CCR2 + monocytes in the border region at all post-MI days, and an increase at day 7 in the remote region, potentially reflecting a secondary inflammatory response in the remote area (21). At all post-MI days, infarct size was similar as assessed by Mac-2 staining (Supplementary Figure S3).

### 3.3. Macrophages reprogram toward glycolysis early after MI

**Figure 2:**
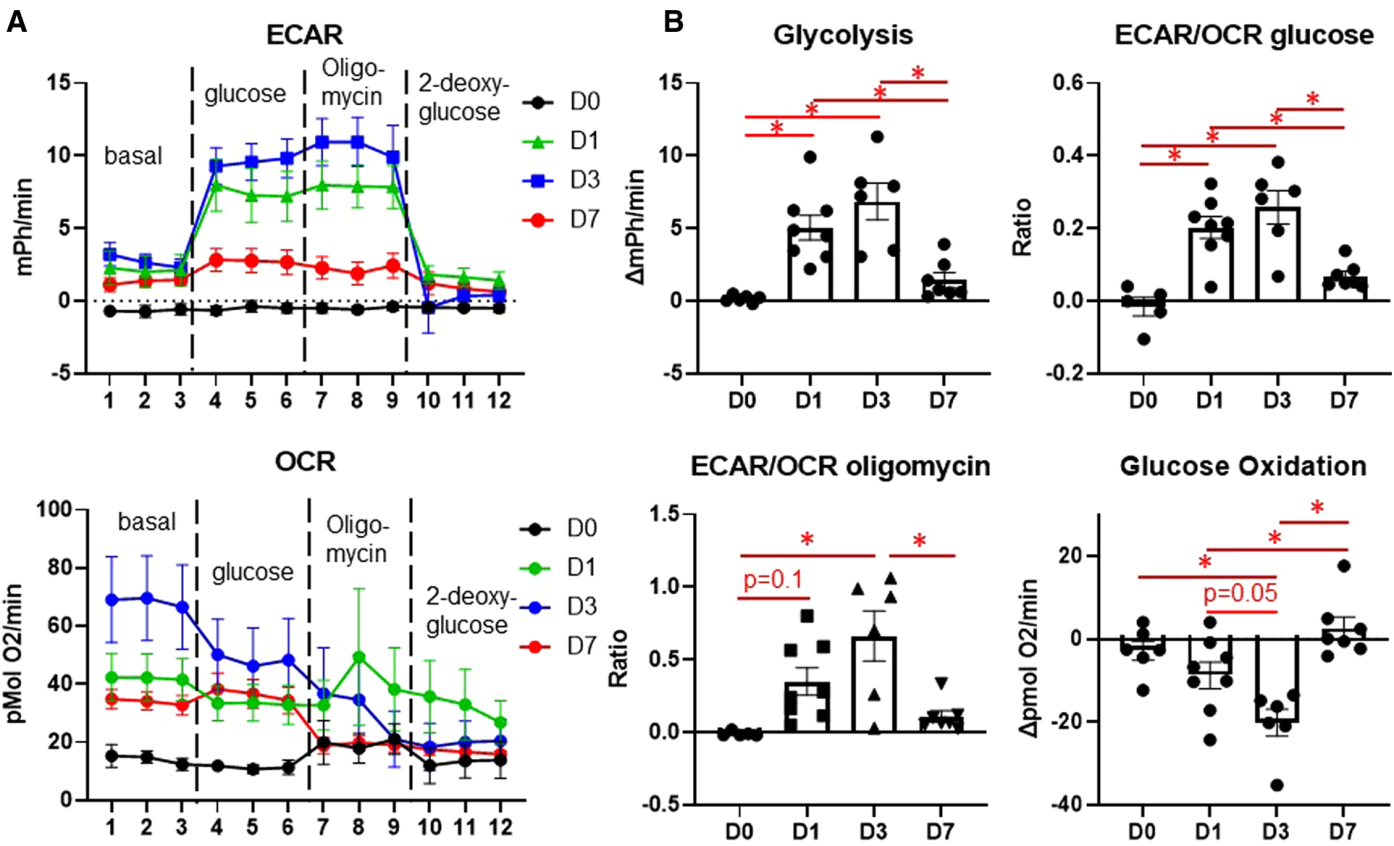
Changes in glucose metabolism in cardiac macrophages after MI. (**A**) Representative Seahorse plots for ECAR (top) and OCR (bottom) for glycolysis stress test. Dashed lines indicate time point at which each stimulation began. (**B**) Quantification of glycolysis, ECAR/OCR upon glucose exposure, glucose oxidation, and ECAR/OCR upon oligomycin exposure. ECAR—extracellular acidification rate, OCR—oxygen consumption rate. *p < 0.05, *n* = 6–8 per group.

We then assessed temporal changes in macrophage glucose metabolism at days 0, 1, 3, and 7 post-MI by extracellular flux analysis. Macrophages showed increased glycolysis at days 1 and 3, as well as increased ECAR/OCR, indicating preference on glycolysis over mitochondrial OXPHOS (Figure 2A). Day 3 macrophages also showed a higher ECAR/OCR after oligomycin stimulation, indicating a higher maximal glycolytic response (16). At day 7, however, glycolysis returned to basal (day 0) levels. Day 3 macrophages also showed a significant decrease in glucose oxidation (Figure 2B) compared to day 0 and day 1 (*p* = 0.05), which returned to basal (day 0) levels by day 7. These data indicate that metabolic reprogramming towards glycolysis occurs during the early phases of MI, i.e., when monocytes predominate, while reverting towards an oxidative phenotype during the later wound healing phase when anti-inflammatory macrophages predominate.

### 3.4. Macrophage gene expression reflects changes in glycolysis, TCA cycle, and PPP

**Figure 3:**
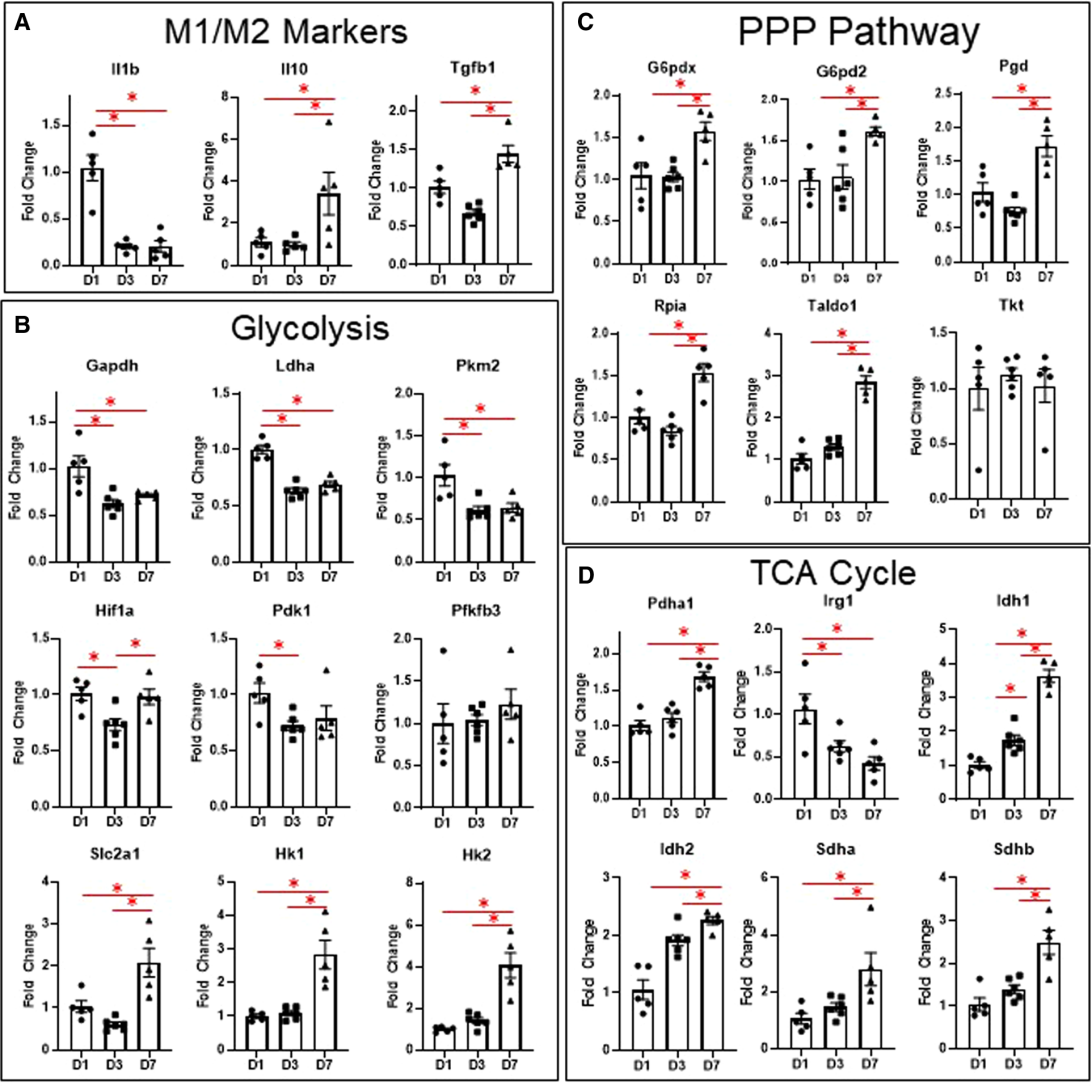
Changes in macrophage gene expression at different time points after MI. (**A**) M1/M2 markers. (**B**) Genes relates to glycolysis. (**C**) Genes related to the PPP pathway. (**D**) Genes related to the TCA cycle. PPP—pentose phosphate pathway. TCA—tricarboxylic acid cycle. *p < 0.05, *n* = 5–6 per group.

To corroborate our results from flow cytometry and live metabolic flux, we isolated macrophages from the infarct area at days 1, 3, and 7 and measured changes in genes involved in glycolysis, the TCA cycle, and the PPP, as well as genes associated with M1/M2 polarization (Figure 3). Due to the large number of mice required to pool cardiac macrophages and produce quality RNA at day 0 (3), we excluded day 0 mice from the gene expression analysis. For more detailed analysis of gene expression analysis of infarct macrophages compared to day 0 controls, please refer to our previous study (3). As expected, we found that *Il1b*, the major driver of post-MI inflammation (3), was increased in macrophages at day 1 relative to days 3 and 7, while *Il10* and *Tgfb1*, which are M2 cytokines (4, 22, 23), were increased at day 7 (Figure 3A). These data reflect our flow cytometry results in which pro-inflammatory monocytes are elevated at day 1, while anti-inflammatory macrophages are increased at day 7.

Several glycolytic genes were also elevated in macrophages at day 1 (Figure 3B), including *Gapdh*, *Pkm2*, and *Ldha*. We also measured expression of glycolytic genes, including *Slc2a1*, *Hk1*, *Pfkfb3*, *Pfkm*, *Pkm*, *Ldha*, and *Pdha1* in the remote (non-infarcted) region of the LV at days 1, 3, and 7 after MI (Supplementary Figure S4). Both *Slc2a1* and *Pfkfb3* were increased in the remote LV at day 1, while *Pfkm* was decreased at day 7 (compared to day 0). *Pkm* was also decreased at day 7 compared to day 1 and 3. These data reflect the increases in macrophage glycolytic metabolism observed by Seahorse during the early phases after MI.

In infarct macrophages, *Hif1a* was increased at day 1 relative to day 3, but was not different at day 7 relative to day 1 (Figure 3B). Expression of *Vegfa*, a major HIF-1α-response gene, showed a similar pattern of expression (Supplementary Figure S5). No differences in *Hif2a*, which has been reported to suppress macrophage mitochondrial metabolism after MI (15), were observed (Supplementary Figure S5).

To our surprise, *Slc2a1*, the major glucose transporter, as well as *Hk1* and *Hk2*, whose enzyme products phosphorylate glucose to glucose-6-phosphate, were elevated in macrophages at day 7 (Figure 3B). Since this did not reflect the decreased expression of the other glycolytic genes at day 7, we tested for expression of genes associated with the PPP, another branch of glucose metabolism that branches off at glucose-6-phosphate (13, 24). We found that of the 6 genes tested, 5 were significantly elevated (Figure 3C, *G6pd2*, *G6pdx*, *Pgd*, *Rpia*, *Taldo1*), indicating increased PPP activity at day 7. By LC-MS, ribose-5-phosphate levels were also significantly elevated at day 7 (Supplementary Figure S6). Since increased PPP activity has been associated with metabolism of heme (25), we measured expression of heme oxygenase 1 (*Hmox1*, Supplementary Figure S5) but observed no differences.

In terms of the TCA cycle (Figure 3D), we found that *Pdha1* was elevated at day 7, which encodes for pyruvate dehydrogenase E1-alpha subunit, reflecting the increase in glucose oxidation we observed by Seahorse analysis at day 7. *Irg1*, which is induced by inflammatory stimuli and produces itaconate from cis-aconitate (26), was increased at day 1. *Idh1* and 2, whose enzyme products convert isocitrate to alpha-ketoglutarate, an anti-inflammatory metabolite (11), were both elevated at day 3 and further at day 7. *Sdha* and b, which convert succinate to fumarate, thus decreasing availability of the pro-inflammatory succinate metabolite (14), were elevated at day 7. These data indicate that changes in TCA cycle intermediates may contribute to pro- and anti-inflammatory macrophage phenotypes after MI.

Overall, these data reflect our Seahorse results and support a role for increased glycolytic metabolism during the early inflammatory phase, while indicating that glucose oxidation/mitochondrial TCA cycle are associated with the M2 phenotype during the wound healing phase. Our results also suggest that the PPP may be uncoupled from glycolysis.

### 3.5. Metabolic reprogramming is attenuated in CCR2- resident macrophages after MI

To compare metabolic reprogramming between infiltrating monocytes and resident macrophages, we used mice lacking the *Ccr2* gene, which possess monocytes that are incapable of extravasating into the infarcted myocardium (27, 28). We compared the response of these mice to C57BL/6J controls at days 1 and 3 after MI. Compared to C57BL/6J controls, there was a decrease in LVIDd and LVIDs in CCR2 KO mice at day 1, but otherwise no major differences in morphological or echocardiographic parameters were observed at days 1 or 3 (Supplementary Table S3). As expected for these acute time points, we observed no mortality in either the C57BL/6J control or CCR2 KO group at either day 1 or day 3.

**Figure 4:**
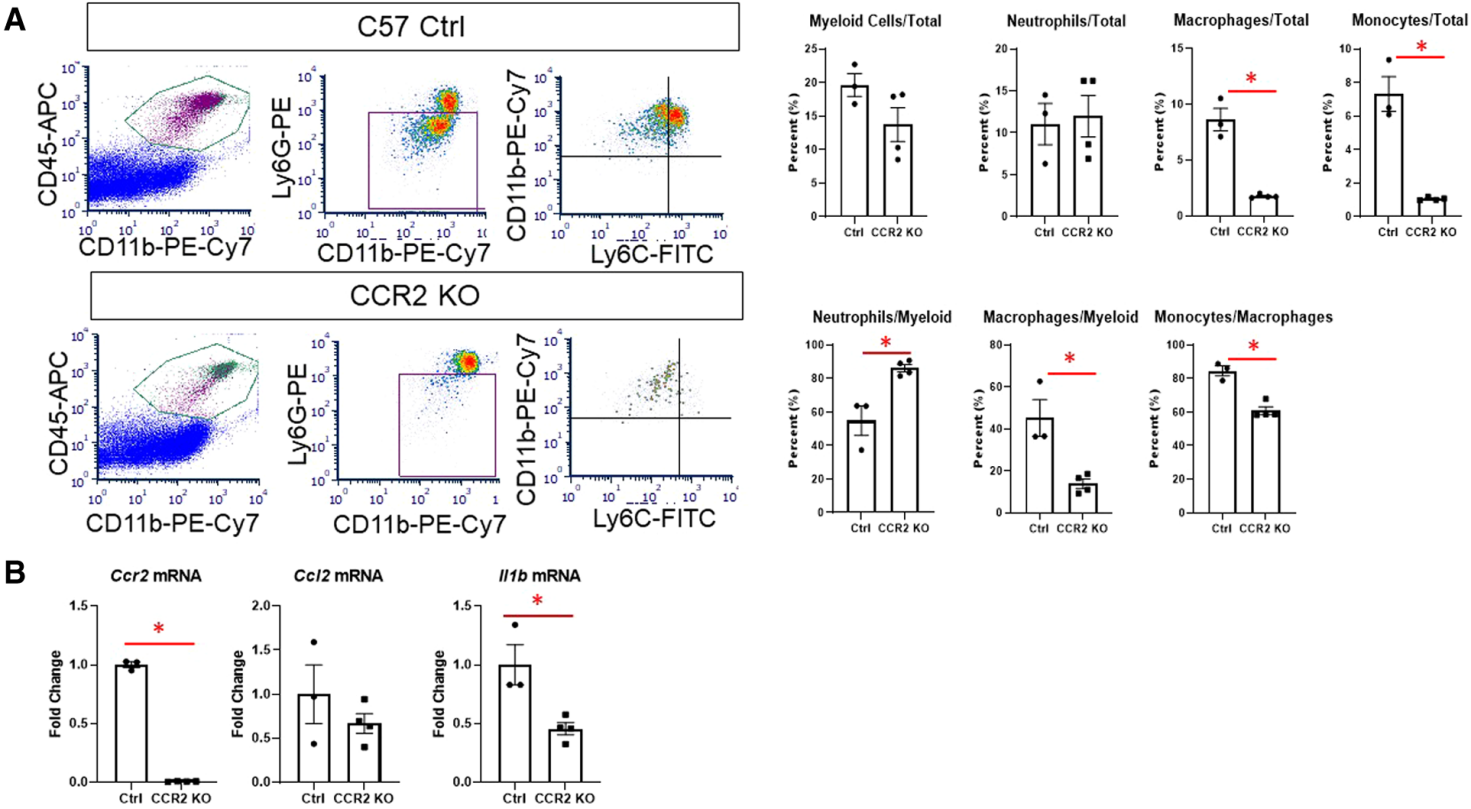
Flow cytometry assessment of cardiac immune cells and inflammatory markers after MI in CCR2 KO mice. (**A**) Representative flow plots for detecting myeloid (CD45 + CD11b+), macrophages (CD11b + Ly6G-) and neutrophils (CD11b + Ly6G+), and monocytes (Ly6C^high^) at day 1 after MI; and quantification of immune cell subtypes. (**B**) Gene expression in the LV infarct tissue at day 1 after MI. *p < 0.05, *n* = 3–4 per group.

We then assessed whether hearts from CCR2 KO mice displayed altered immune cell infiltration at day 1 after MI by flow cytometry. As expected, there were significantly fewer macrophages (CD11b + Ly6G-) in the infarct as expressed by percentage of total cells and percent of total myeloid cells (Figure 4A). There were also fewer total monocytes (CD11b + Ly6G-Ly6C^high^), and a smaller proportion of macrophages were of the monocyte lineage in CCR2 KO mice. There were no differences in neutrophil populations, indicating that the lack of CCR2 only affected monocyte influx. By immunofluorescence, we confirmed that total numbers of Mac-2 + macrophages were decreased in CCR2 KO hearts at post-MI day 3, although as a percentage of total cells were not statistically different from controls (Supplementary Figure S7), indicating that infarct macrophages were not replaced by other cells in CCR2 KO mice. Similar results were observed for CD206+ (M2) macrophages, and as expected, there were no CCR2 + cells in the hearts of CCR2 KO mice. Infarct sizes were similar between control and CCR2 KO mice (Supplementary Figure S8). After confirming that *Ccr2* expression was decreased (Figure 4B), we found that *Il1b* was also significantly decreased in the day 1 infarct, reflecting the decrease in monocyte infiltration (3). We found no changes in expression of *Ccl2*, the major chemokine ligand for CCR2, indicating that the decrease in monocyte infiltration was not due to lack of CCL2 interaction with CCR2.

**Figure 5:**
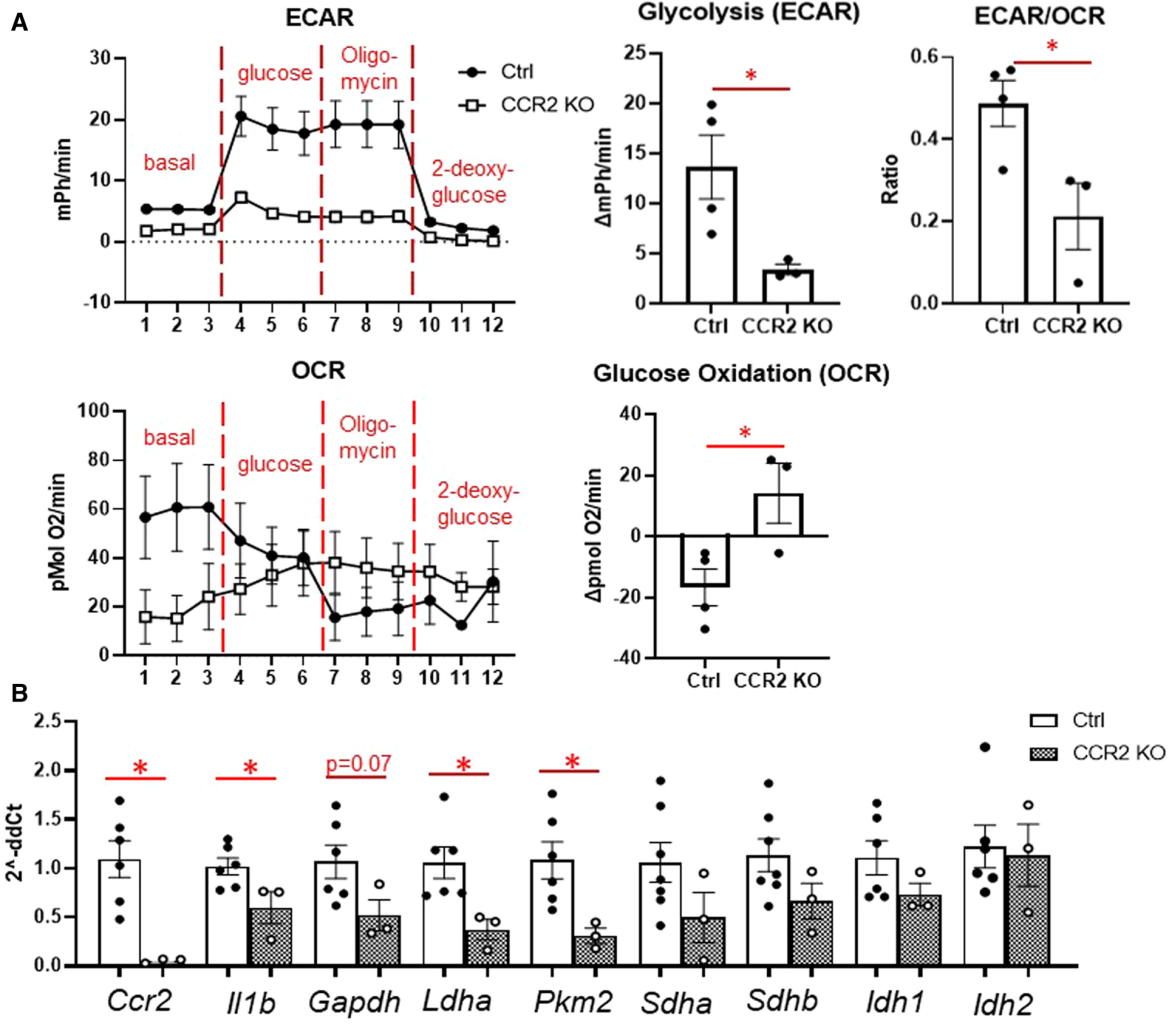
Changes in glucose metabolism in cardiac macrophages after MI in CCR2 KO mice. (**A**) Left—representative Seahorse plots for ECAR and OCR in cardiac macrophages at day 3 after MI, and quantification of glycolysis, ECAR/OCR, and glucose oxidation. ECAR—extracellular acidification rate; OCR—oxygen consumption rate. (**B**) Inflammatory genes (Ccr2 and Il1b), glycolysis (Gapdh, Ldha, Pkm2) and TCA cycle (Sdha and Sdhb, Idh1 and Idh2) were measured. *p < 0.05, *n* = 3–6 per group.

We then assessed macrophage glycolysis and glucose oxidation at day 3 after MI (Figure 5). While monocytes peak at day 1, we chose day 3 to assess macrophage metabolism as maximum increases in glycolysis and decreased glucose oxidation were observed at this time point. Glycolysis and ECAR/OCR was significantly decreased in macrophages from CCR2 KO mice (Figure 5A), while glucose oxidation was significantly increased. In line with these findings, we found that *Il1b*, *Ldha,* and *Pkm2* levels were significantly decreased in isolated infarct macrophages from CCR2 KO mice, while there were no changes in the TCA cycle genes *Sdha/b* and *Idh1/2* (Figure 5B). These results indicate that glycolytic metabolic reprogramming is a feature of monocyte-derived but not resident macrophages.

### 3.6. Dichloroacetate administration did not alter macrophage phenotype or metabolism after MI

**Figure 6:**
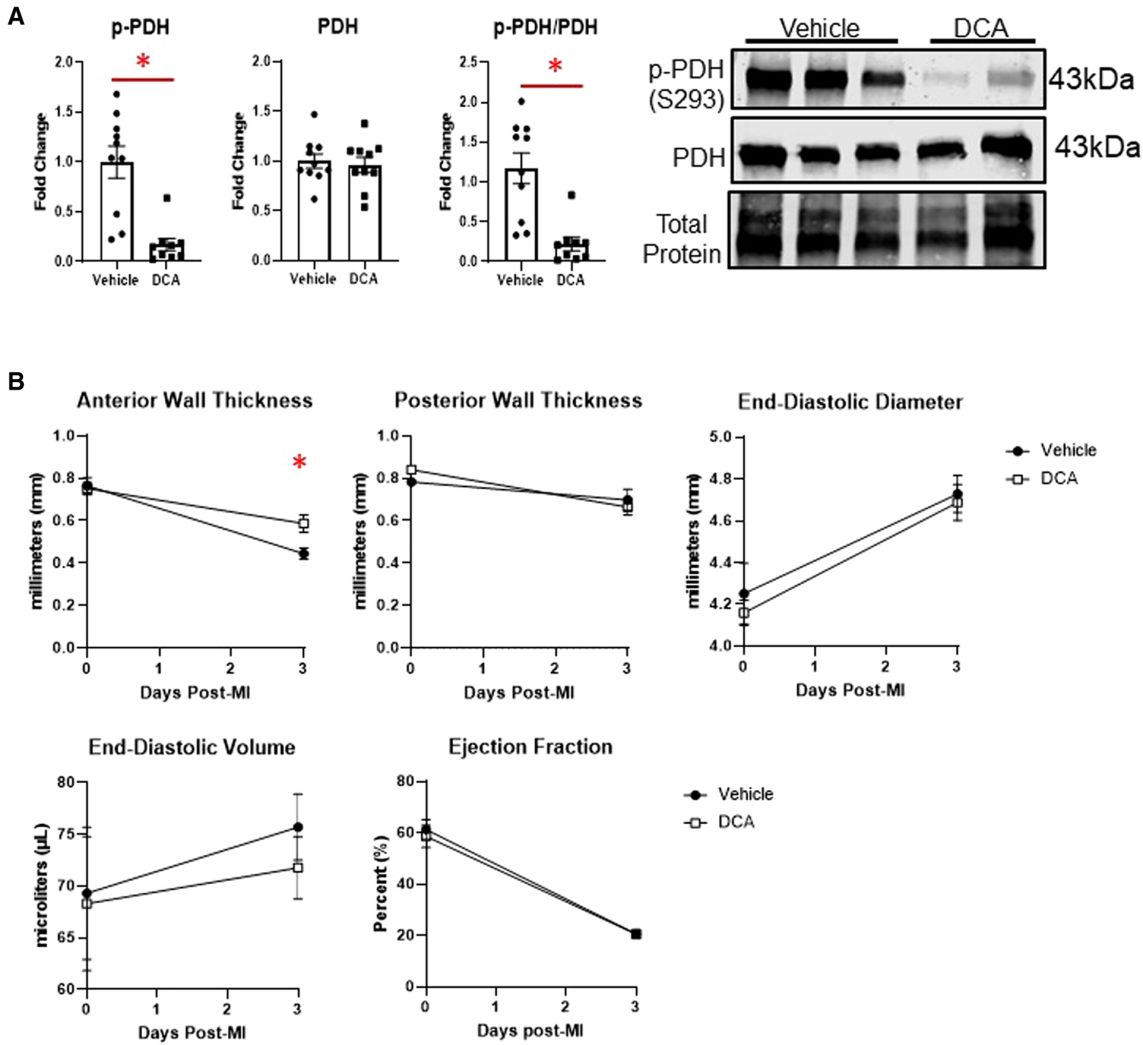
Effects of dichloroacetate (DCA) on cardiac function after MI. (**A**) DCA significantly decreased inhibitory phosphorylation of PDH (S293), indicating PDH activation. (**B**) DCA attenuated anterior wall thinning at day 3 post-MI, but did not affect posterior wall thickness, end-diastolic diameter or volume, or ejection fraction. *p < 0.05, *n* = 9–10 per group.

**Figure 7:**
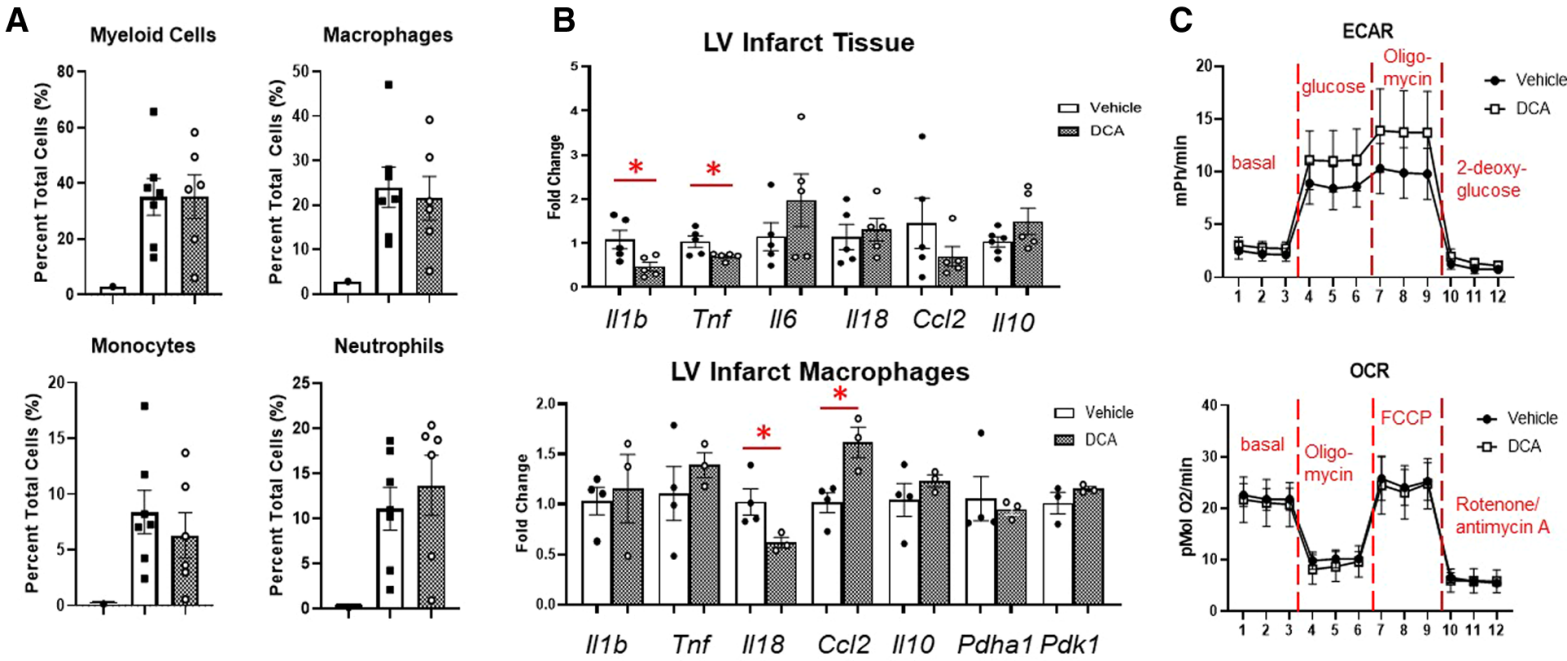
Effects of DCA on post-MI inflammation and macrophage phenotype. (**A**) DCA did not affect immune cell infiltration into the heart as assessed by flow cytometry. (**B**) DCA decreased *Il1b* and *Tnf* mRNA in the LVI tissue. In LV infarct macrophages, DCA decreased *Il18*, and increased *Ccl2*. (**C**) DCA did not affect macrophage glycolysis or OXPHOS. *p < 0.05. *n* = 6–7 per group.

To assess whether we could target macrophage metabolism by pharmacological means, we administered dichloroacetate (DCA), a PDK1 inhibitor, for up to 3 days after MI. PDK1 phosphorylates PDH at serine 293 to inhibit its activity (18). DCA administration resulted in robust de-phosphorylation of PDH at serine 293, indicating PDH activation (Figure 6A). By echocardiography, DCA attenuated thinning of the anterior wall (Figure 6B), but did not affect posterior wall thickness, end-diastolic diameter or volume, or ejection fraction. No differences in infarct size were observed between vehicle and DCA-treated mice (Supplementary Figure S9). DCA did not affect total numbers of myeloid cells, macrophages, monocytes, or neutrophils as assessed by flow cytometry (Figure 7A), or Mac-2+, CD206+, or CCR2 + macrophages by immunofluorescence (Supplementary Figure S10). However, DCA decreased LV infarct levels of *Il1b* and *Tnf* mRNA, while not affecting *Il6*, *Il18*, *Ccl2*, or *Il10* (Figure 7B). In isolated macrophages however, DCA did not affect *Il1b*, *Tnf*, *Il10*, *Pdha1*, or *Pdk1*, but did decrease *Il18* while elevating *Ccl2* (Figure 7B). Finally, DCA did not affect macrophage glycolysis or OXPHOS (Figure 7C), suggesting that the macrophage immunometabolic phenotype may not be regulated by Pdk1.

## 4. Discussion

Our results indicate that macrophages exhibit temporal glucose metabolic reprogramming that reflects their polarization status between monocyte-derived pro-inflammatory and anti-inflammatory/pro-reparative subtypes. Furthermore, our results show that resident CCR2- macrophages do not undergo metabolic reprogramming towards glycolysis early after MI, indicating that major shifts in glucose metabolism may be unique to monocyte-derived macrophages. Another major finding of our study was that despite a reversion of the glycolytic phenotype at day 7, glucose uptake and pentose phosphate pathway (PPP) genes were markedly increased. These data suggest that glucose metabolism plays a critical role during macrophage polarization after MI, particularly in monocytes.

### 4.1. Temporal changes in macrophage glucose metabolism and gene expression after MI

Metabolic reprogramming is a hallmark of macrophage polarization (11, 12, 15, 16, 23, 24, 29). The canonical metabolic reprogramming signature of M1 macrophages is an increase in anaerobic glycolysis, increased lactate production, and attenuated TCA cycle activity and OXPHOS; while M2 macrophages display decreased glycolysis, full TCA cycle activity, and increased reliance on glucose and fatty acid oxidation (14, 30). In the infarcted heart, the role of macrophage glycolysis has been highlighted by uptake of radioactive tracers such as ^18^fluorodeoxyglucose, as well as [1-C^13^]-pyruvate which is metabolized to [1-C^13^]-lactate that can be imaged by magnetic resonance (31–33). Our previous work demonstrated that treatment with the immunometabolic agent dimethyl fumarate attenuated post-MI macrophage metabolic reprogramming and inflammation, and improved cardiac remodeling, indicating that targeting macrophage metabolism is a promising therapeutic target (16). In this study, we found similar increases in glycolysis in macrophages at day 1, when inflammation peaks, and day 3, when granulation tissue starts to form (3). Interestingly, glucose oxidation was only decreased at day 3, although the ECAR/OCR ratio was similar to day 1. In line with these findings, glycolytic genes were elevated at day 1, including *Gapdh*, *Pkm2*, and *Ldha*.

In terms of pyruvate metabolism, *Pdha1*, which encodes for the pyruvate dehydrogenase E1-alpha subunit that converts pyruvate to acetyl CoA for entry into the TCA cycle, was elevated at day 7, reflecting the increased glucose oxidation at this time point. The TCA cycle genes *Sdha* and *Sdhb*, which encode for succinate dehydrogenase (SDH), were both elevated at day 7. SDH catalyzes the conversion of succinate to fumarate, thereby decreasing the levels of the pro-inflammatory succinate (29). SDH is one of the major break points of the TCA cycle in M1 macrophages, allowing succinate to accumulate and activate HIF-1α (14, 29). *Idh1* and *Idh2,* which encode for isocitrate dehydrogenase (IDH), were both elevated at day 3 and day 7. IDH catalyzes the conversion of isocitrate to alpha-ketoglutarate, which is an anti-inflammatory metabolite that promotes M2 polarization (11, 14). Thus, changes in TCA metabolites such as succinate and alpha-ketoglutarate may reflect metabolic reprogramming and influence macrophage polarization after MI.

While our gene expression data closely reflected the changes in glucose metabolism observed by Seahorse analysis in day 1 and 7 macrophages, day 3 macrophages displayed a gene expression profile more similar to day 7 macrophages in terms of glycolytic and TCA cycle genes. Day 3 macrophages represent a transitionary phase between day 1 and 7, as resident macrophages begin to migrate/proliferate and contribute to the overall pool of macrophages, and the number of M1/M2 macrophages is approximately equal (3, 10). Our previous study using transcriptomics demonstrated that in day 3 macrophages, glycolytic genes revert to basal levels while mitochondrial genes are highly expressed (3), suggesting that while metabolic reprogramming appears to peak at day 3, changes in the transcriptional profile may occur to accommodate the rapid change in phenotype.

### 4.2. Role of the pentose phosphate pathway in post-MI macrophage metabolic reprogramming

While many of our results confirmed our notions about canonical macrophage metabolism, our finding that the PPP was activated at day 7, at which point the majority of macrophages are of the M2 phenotype and glycolytic metabolism has reverted back to a basal phenotype, was surprising. Activation of the PPP is a hallmark of M1-polarized glycolytic macrophages, as the PPP provides NADPH as a critical co-factor for reactive oxygen species (ROS)-producing enzymes, anti-oxidant systems such as glutathione, biosynthesis of pro-inflammatory lipid mediators, and nucleotide synthesis (13). The PPP is typically activated in parallel with increased glycolysis (14); however, our results indicate that in day 7 macrophages, the PPP is upregulated independently of glycolysis.

The PPP is divided into the oxidative branch, which produces NADPH, and the non-oxidative branch, which produces 5-carbon sugars for nucleotide synthesis (13, 24). Previous studies indicate that the oxidative branch is selectively increased in M1 macrophages (34), while the non-oxidative branch may be required for M2 activation (24). We found that genes for both the oxidative (*G6pdx*, *G6pd2*, *Pgd*) and the non-oxidative branch (*Rpia*, *Taldo1*) were increased, indicating increased activity of both branches. As there are relatively few studies investigating the role of the PPP in macrophages, which is typically thought to support M1 polarization, we can only speculate as to the role of the PPP in post-MI day 7 macrophages. One possibility is that PPP intermediates such as ribose-5-phosphate, which was elevated at day 7, is used for biosynthetic pathways during cell proliferation (14), as macrophages reach their peak number by day 7 (10). Another possibility is that day 7 macrophages participate in heme clearance, as heme uptake and metabolism by macrophages diverts glucose away from bioenergetics pathways and towards the PPP (25). Indeed, IL-10, an M2 cytokine, upregulates CD163 in macrophages to promote efficient heme clearance in the heart after MI (22). Since we observed increased expression of *Il10* at day 7, it is possible that increased PPP activation supports heme clearance. However, the role of the PPP in macrophages after MI warrants further investigation. Unfortunately, pharmacological inhibitors for the PPP such as 6-aminonicotinamide are highly toxic to the central nervous system, posing a roadblock to understanding the role of the PPP during MI. Any studies directly investigating the role of the PPP in macrophages should thus involve the use of transgenic animals.

### 4.3. Role of the HIF-1α in post-MI macrophage metabolic reprogramming

HIF-1α is strongly induced in M1 macrophages, including post-MI macrophages at day 1 as a response to hypoxia (3, 12, 15). HIF-1α is considered a master regulator of metabolic reprogramming in macrophages by driving expression of glycolytic genes, particularly *Slc2a1*, the major glucose transporter (12). We found that *Hif1a* was decreased at day 3 compared to days 1 and 7, but showed no difference between day 1 and day 7. By day 3 after MI, formation of granulation tissue occurs, which is characterized by angiogenesis and restoration of blood flow to the infarction region (4, 5). This explains why *Hif1a* would be decreased at day 3 relative to day 1.

Although the finding that *Hif1a* levels are equal in day 1 and 7 macrophages is inconsistent with the M1/M2 paradigm, previous reports demonstrate that sustained increases in HIF-1α are observed in macrophages for up to 30 days after MI, even further increasing at day 30 relative to day 7 (15). Thus, while early activation of HIF-1α may mediate glycolytic metabolism at day 1, other factors may override HIF-1α at later time points to promote downregulation of glycolysis and increased glucose oxidation/mitochondrial TCA cycle. As explained in the previous section, one of our major findings was that in day 7 macrophages, Slc2a1 and Hk1/2, which mediate glucose uptake and phosphorylation, were increased, indicating that day 7 macrophages increase glucose uptake to support the PPP rather than glycolysis. One intriguing possibility is that HIF-1α stays elevated at day 7 to support an increase in glucose uptake, as *Slc2a1* is a HIF-1α target gene and was upregulated at day 7. However, further studies are needed to assess the role of HIF-1α in macrophage metabolic reprogramming and PPP.

### 4.4. Role of glucose metabolism in resident vs. monocyte-derived macrophages

To investigate differences in metabolic reprogramming between monocytes and resident cardiac macrophages, we used mice lacking the Ccr2 gene (CCR2 KO), which is the endogenous receptor for the CCL2 chemokine (7, 8, 20, 28). The majority of resident cardiac macrophages are CCR2- and mediate anti-inflammatory/pro-reparative functions during cardiac injury, while CCR2+ macrophages are derived from peripheral monocytes and are highly pro-inflammatory (8, 20). During cardiac injury, however, CCR2+ infiltrate the heart and replace CCR2- macrophages (7, 28). Our results showed that at day 1, CCR2 KO mice had substantially fewer infarct macrophages than controls, and these were of the resident macrophage phenotype (Ly6C^low^). As resident CCR2- macrophages are anti-inflammatory, it was unsurprising that these resident macrophages exhibited lower glycolysis and increased glucose oxidation. While glycolytic genes were less expressed in CCR2- macrophages, SDH and IDH isoforms were not elevated, despite increased glucose oxidation. This could be due to inherently different levels of these genes in macrophages of different origin (3). Another possibility is that resident macrophages use less of other substrates that feed into the TCA cycle, such as fatty acids (23). Overall, our data suggest that metabolic reprogramming is a unique feature of monocyte-derived macrophages. It is possible that an inability to use glycolysis may impair the ability of resident macrophages to survive a hypoxic environment, as the majority of resident macrophages are killed off very early after ischemia (7).

### 4.5. Role of dichloroacetate in targeting macrophage metabolism

To target immunometabolism pharmacologically, we used the drug dichloroacetate (DCA), a pyruvate dehydrogenase kinase (PDK) inhibitor. Targeting macrophage metabolism *in vitro* with DCA prevents the inhibitory actions of PDK on the pyruvate dehydrogenase complex, which increases oxidation of pyruvate derived from glycolysis and decreases the reliance on anaerobic glycolysis (12, 35–37). DCA has also been observed to improve cardiac outcomes after myocardial ischemia/reperfusion injury (18, 38), but its role in modulating immune cell function after MI has not been investigated. While we found that DCA decreased LV infarct wall thinning and decreased infarct expression of the pro-inflammatory cytokines *Il1b* and *Tnf*, it did not affect macrophage expression of these cytokines. While macrophages are major sources of *Il1b* and *Tnf* in the heart, other cell types can produce these cytokines in response to injury, including neutrophils, myocytes, fibroblasts, and endothelial cells (39–41), suggesting that DCA perhaps decreased cytokine expression from another cell source. DCA did decrease *Il18* but increased *Ccl2*, suggesting that DCA overall did not skew macrophage polarization towards a particular phenotype.

### 4.6. Further considerations

Another finding of our study is the metabolic remodeling of other cell types in the heart, which may be affected by interventions targeting glycolytic metabolism. For example, *Slc2a1* and *Pfkfb3* were elevated in the remote region at day 1, indicating that myocytes may increase glycolytic metabolism acutely after MI. On the other hand, *Pfkm* and *Pkm* were decreased at day 7, indicating that the remote region may become less glycolytic as the heart undergoes further remodeling and repair. Although the exact role of glycolysis after MI is controversial, several studies have suggested that enhanced glycolysis is beneficial for maintaining contractile function (42–44), although this may require the full oxidation of glucose (45, 46).

While our data suggest that monocytes undergo metabolic reprogramming after infiltrating the heart, a question that our study did not address is whether monocytes reprogram toward glycolysis prior to infiltrating the infarcted tissue, which could support key functions such as mobilization at extra-cardiac sites and migration to the injured area (12). Another question is whether monocytes switch from glycolysis to OXPHOS by day 7, or whether these monocytes are simply replaced with resident macrophages, which are already more oxidative to begin with. Since both resident and monocyte-derived macrophages ultimately give rise to reparative macrophages during the wound healing phase (6, 47), it would be interesting to determine whether these two lineages show similar metabolic reprogramming at later time points.

### 4.7. Limitations

Our study has some limitations. The most obvious limitation is that our isolation method captures a total pool of macrophages from the heart (3). However, as indicated in our discussion, cardiac macrophages are heterogenous and exist in several different subsets of populations, the most distinct being resident vs. monocyte-derived (7, 20). These can be further subdivided into populations based on several markers, most notably MHC II, which imparts antigen-presenting activity (7). While day 1 macrophages can be broadly identified as “M1-like” and day 7 as “M2-like,” individual differences between these populations based on cell surface markers and secreted cytokines cannot be ignored (10). Furthermore, for our gene expression analysis, we only compared macrophages from day 1, 3, and 7 mice, and excluded day 0 as a control. Thus, future studies should seek to understand time-dependent differences in metabolic reprogramming after cardiac injury.

Our study analyzed macrophages isolated at different time points after MI. An important question that arises from our analysis is whether metabolic reprogramming occurs within individual macrophages over time, or whether glycolysis-preferring cells (i.e., monocytes) are replaced by OXPHOS-preferring cells (i.e., resident M2 macrophages). The answer is likely both, as the source of M2 macrophages during the scar maturation phase is a mixture of both differentiated monocytes and proliferation of local resident macrophages (6, 20, 48). Future studies should employ lineage tracing to fully assess whether metabolic reprogramming occurs in the same cells to account for different sources of macrophages.

We focused on macrophage glucose metabolism, as glycolysis is known to be a critical regulator of M1 macrophage function and relies solely on glucose as an energy source (14). However, other fuel types, such as fatty acids and amino acids, are important as well. For example, efferocytosis of necrotic myocytes supplies macrophages with fatty acids that supply mitochondrial respiration and generate NAD+ to drive M2 polarization (23). However, the roles of other fat-derived fuels such as ketone bodies, which have anti-inflammatory properties (49), and amino acids such as glutamine (50) in driving metabolic reprogramming and macrophage polarization after MI are unknown and warrant further investigation.

We used CCR2 KO mice to study the role of resident vs. monocyte-derived macrophages. This model has been used to block monocytes from infiltrating the heart during injury, and thus investigate differential roles of CCR2+ monocytes and CCR2- resident macrophages during cardiac remodeling (28). One potential limitation of this model is that in the absence of infiltrating monocytes, resident macrophages may display a different phenotype in the infarcted heart. The presence of inflammatory monocytes could also alter the microenvironment to which resident macrophages are exposed, such as changes in extracellular matrix architecture, vascularity, and cytokine gradients (9). Our results found a significant decrease in infarct *Il1b* mRNA in CCR2 KO mice, indicating that resident macrophages are likely exposed to a different inflammatory milieu than in control mice. Whole body deletion of *Ccr2* does not appear to affect the anti-inflammatory/pro-reparative phenotype of CCR2- resident macrophages (7, 27). However, future studies should attempt to assess metabolism of both resident and monocyte-derived macrophages from the same heart to fully answer this question.

A final limitation is that our study used only male mice. Given that there are sex differences in macrophage roles following MI (51), future studies in immunometabolic macrophage phenotypes should explore potential sex differences.

In conclusion, our data suggest an important role for glucose metabolism in macrophage activation and polarization following MI. Our data confirm that glycolytic metabolic reprogramming occurs in M1 macrophages during the early inflammatory phase, while reversion to an oxidative phenotype was found in M2 macrophages during the later phase of wound healing and resolution of inflammation. Paradoxically, PPP genes are activated in M2 oxidative macrophages, indicating a potential role for the PPP in M2 polarization after MI. Finally, metabolic reprogramming was observed in monocyte-derived but not resident macrophRages. Our results suggest that glycolysis may be a potential therapeutic target for limiting early monocyte-mediated inflammation, though the potential adverse effects on the PPP should be taken into consideration.

## Data Availability

The original contributions presented in the study are included in the article/**Supplementary Materials**, further inquiries can be directed to the corresponding author/s.
