# Supplementary material for: Temporal changes in glucose metabolism reflect polarization in resident and monocyte-derived macrophages after myocardial infarction
Source: Front Cardiovasc Med. 2023 May 5;10:1136252. doi: 10.3389/fcvm.2023.1136252 (PMC10196495; doi:10.3389/fcvm.2023.1136252)
Supplement: Supplementary file 1 [file Table1.docx]

***Supplemental Materials***

***Liquid Chromatography Mass Spectrometry (LC-MS).***

Frozen samples were stored at -80°C until analyzed by LC-MS-based metabolomics in the Vanderbilt Center for Innovative Technology (CIT). Isotopically labeled phenylalanine-D8 and biotin-D2 were added to 200 μL of culture supernatant per sample, and protein was precipitated by addition of 800 μL of ice-cold methanol followed by overnight incubation at −80°C. Precipitated proteins were pelleted by centrifugation (15,000 rpm, 15 min), and extracted metabolites were dried down in vacuo and stored at −80°C. Individual samples were reconstituted in 100 μL of reconstitution buffer (acetonitrile/water, 90:10, vol/vol) containing tryptophan-D3 and inosine-4N15. Equal volumes of individual samples were pooled to create a quality control (QC) pooled sample used for column conditioning, retention time alignment, to assess instrument reproducibility, and for batch acceptance.

LC-MS and LC-MS/MS analyses were performed on a high-resolution Q-Exactive HF hybrid quadrupole-Orbitrap mass spectrometer (Thermo Fisher Scientific, Bremen, Germany) equipped with a Vanquish UHPLC binary system and autosampler (Thermo Fisher Scientific, Germany). Metabolite extracts were separated on ACQUITY UPLC BEH Amide HILIC 1.7 μm, 2.1 × 100 mm column (Waters Corporation, Milford, MA) held at 30°C. Liquid chromatography was performed at a 200 μL min using solvent A (5 mM Ammonium formate in 90% water, 10% acetonitrile, and 0.1% formic acid) and solvent B (5 mM Ammonium formate in 90% acetonitrile, 10% water, and 0.1% formic acid) with a gradient length of 30 min.

Full MS analyses (8 μL injection volume) were acquired over 70 to 1,050 mass-to-charge ratio (m/z) in negative ion mode. Full mass scan was acquired at 120K resolution with a scan rate of 3.5 Hz, automatic gain control (AGC) target of 10e6, and maximum ion injection time of 100 ms. MS/MS spectra were collected at 15K resolution, AGC target of 2e5 ions, and maximum ion injection time of 100 ms.

The acquired raw data were imported, processed, normalized and reviewed using Progenesis QI v.3.0 (Non-linear Dynamics, Newcastle, UK). All MS and MS/MS sample runs were aligned against a QC pool reference run, and unique ions (retention time and *m/z* pairs) were deadducted and deisotoped to generate unique “features” (retention time and *m/z* pairs). Data were normalized to all features using Progenesis QI. Experimental data annotations were assigned based on consistent retention time and MS^2^ fragmentation pattern matches with reference standards.

| **Supplemental Table 1. Primer Sequences.** | |
| --- | --- |
| Gene | Sequence |
| Actb | 5’-GATTACTGCTCTGGCTCCTAG-3’  5’-GACTCATCGTACTCCTGCTTG-3’ |
| G6pdx | 5’-GAAGCAGTCACCAAGAACATTC-3’  5’-TGGTTCGACAGTTGATTGGAG-3’ |
| G6pd2 | 5’-GATCTACCGCATTGACCACTAC-3’  5’-TCCATTCCAGATAGGGCCAA-3’ |
| Hif1a | 5’-GCTCACCATCAGTTATTTACGTG-3’  5’-CCGTCATCTGTTAGCACCAT-3’ |
| Hk1 | 5’-GCTGCCTTCTTATGTTCGGA-3’  5’-CAGTAGGACTCGGAAATTCGTT-3’ |
| Hk2 | 5’-GAACCAGATCTACGCCATTCC-3’  5’-ATCTGTAGCTTGTCCATGAAGT-3’ |
| Idh1 | 5’-GATGTGCAGTCAGACTCAGT-3’  5’-GCTTCTACCGTCTTACCATCTG-3’ |
| Idh2 | 5’-AGTGTGGCTGTCAAGTGTG-3’  5’-AAGGATGTTCCGGATCGTTC-3’ |
| Il1b | 5’-GACCTGTTCTTTGAAGTTGACG-3’  5’-CTCTTGTTGATGTGCTGCTG-3’ |
| Il10 | 5’-GTCATCGATTTCTCCCCTGTG-3’  5’-ATGGCCTTGTAGACACCTTG-3’ |
| Irg1 | 5’-CTACAGTTCCAACACCTCCAG-3’  5’-GCCATGTGTCATCAAAATCCAT-3’ |
| Ldha | 5’-CTCCCCAGAACAAGATTACAGT-3’  5’-CCCTTGAGTTTGTCTTCCATGA-3’ |
| Pdha1 | 5’-CATGAGTGACCCTGGAGTAAG-3’  5’-GCTGTTCACCATTCTATCCTTG-3’ |
| Pdk1 | 5’-ACCAGCACTCCTTATTGTTCG-3’  5’-GCCTAGCGTTCTCATAGCC-3’ |
| Pfkfb3 | 5’-ACTCGCTACCTCAACTGGATA-3’  5’-TCATGGCTTCCTCATTGTCAG-3’ |
| Pgd | 5’-GATCTTCCAAGCCATCGCT-3’  5’-CCATACTCTATCCCGTTGTGC-3’ |
| Pkm2 | 5’-CTGAAGGAGATGATTAAGTCTGGA-3’  5’-TGGCTTCACGGACATTCTTG-3’ |
| Rpia | 5’-GCTTAACCCTCAGTGACCTG-3’  5’-CTCCACCCTTGATGAGATTGAG-3’ |
| Sdha | 5’-TCCATACACCGAATAAGAGCAAA-3’  5’-ACCAGCCCTAGTGAACCAT-3’ |
| Sdhb | 5’-CAGTATCTGCAGTCCATCGAG-3’  5’-TGTCTCCGTTCCACCAGTA-3’ |
| Slc2a1 | 5’-AGTTCGGCTATAACACTGGTG-3’  5’-GTGGTGAGTGTGGTGGATG-3’ |
| Taldo1 | 5’-CGGCAAGGACAGAATTCTCAT-3’  5’-AGTGTCATGTTGCAGTGGAT-3’ |
| Tkt | 5’-GAAACAAGCCTTCACCGATG-3’  5’-CTCCCAGCATGCAATAGACTC-3’ |

| **Supplemental Table 2. Echocardiographic and Morphometric Parameters after MI.** | | | | |
| --- | --- | --- | --- | --- |
|  | **Day 0** | **Day 1** | **Day 3** | **Day 7** |
| EF (%) | 61±2 | 17±1* | 18±1* | 14±1* |
| LVAWd (mm) | 0.91±0.03 | 0.56±0.03* | 0.64±0.07* | 0.41±0.03*#$ |
| LVPWd (mm) | 0.85±0.03 | 0.71±0.03 | 0.83±0.05 | 0.57±0.06*$ |
| LVIDd (mm) | 4.2±0.1 | 4.4±0.1* | 4.3±0.1* | 6.0±0.2*#$ |
| LVEDV (μL) | 62±3 | 83±3* | 80±4 | 133±9*#$ |
| LV mass (mm/mm) | 5.6±0.1 | 6.4±0.1 | 7.4±0.2*# | 7.4±0.2*# |
| Lung mass (mm/mm) | 10.0±0.8 | 10.5±0.3 | 11.0±0.8 | 17.1±1.6*#$ |
| EF—ejection fraction; LVAWd—left ventricular anterior wall thickness; PWd—posterior wall thickness; IDd—internal diastolic diameter; EDV—end-diastolic volume. LV and lung mass normalized to tibia length. *p<0.05 vs Day 0, #p<0.05 vs day 1, $p<0.05 vs day 7. | | | | |

| **Supplemental Table 3. Echocardiographic and Morphological Parameters in CCR2 KO mice after MI.** | | | | | |
| --- | --- | --- | --- | --- | --- |
|  | **C57 Day 1** | **CCR2 KO Day 1** | **C57 Day 3** | **CCR2 KO Day 3** |  |
| EF (%) | 12±3 | 16±2 | 13±2 | 15±1 |  |
| LVAWd (mm) | 0.50±0.08 | 0.48±0.03 | 0.55±0.04 | 0.60±0.04 |  |
| LVPWd (mm) | 0.73±0.14 | 0.77±0.004 | 0.74±0.03 | 0.72±0.05 |  |
| LVIDd (mm) | 4.92+0.17 | 4.54±0.03* | 4.8±0.1 | 4.9±0.1 |  |
| LVIDs (mm) | 4.64±0.03 | 4.29±0.03* | 4.4±0.1 | 4.5±0.1 |  |
| LVEDV (μL) | 81±4 | 77±2 | 75±5 | 75±4 |  |
| Body weight (g) | 26.7±1.3 | 25.0±0.4 | 27.5±1.3 | 26.2±0.3 |  |
| LV mass/tibia (mg/mm) | 6.00±0.07 | 5.54±0.10 | 6.68±0.21 | 6.71±0.18 |  |
| Lung mass/tibia (mg/mm) | 10.1±1.4 | 9.6±0.4 | 10.3±0.6 | 10.4±0.47 |  |
| EF—ejection fraction; LVAWd—left ventricular anterior wall thickness; PWd—posterior wall thickness; IDd—internal diastolic diameter; EDV—end-diastolic volume. LV and lung mass normalized to tibia length. *p<0.05 vs C57 day 1. | | | | | |

**Supplementary Figure 1.**

**Supplementary Figure 1.** Post-MI survival curve for mice used for the day 7 time-point.

**Supplementary Figure 2.**

**
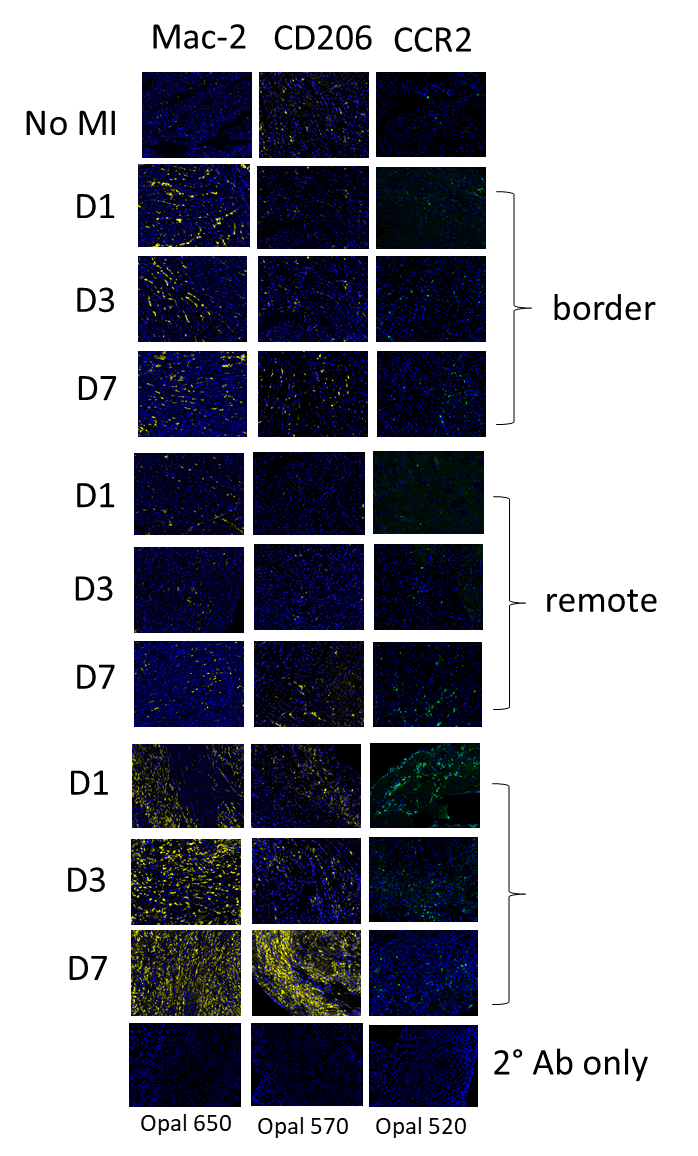
**

**
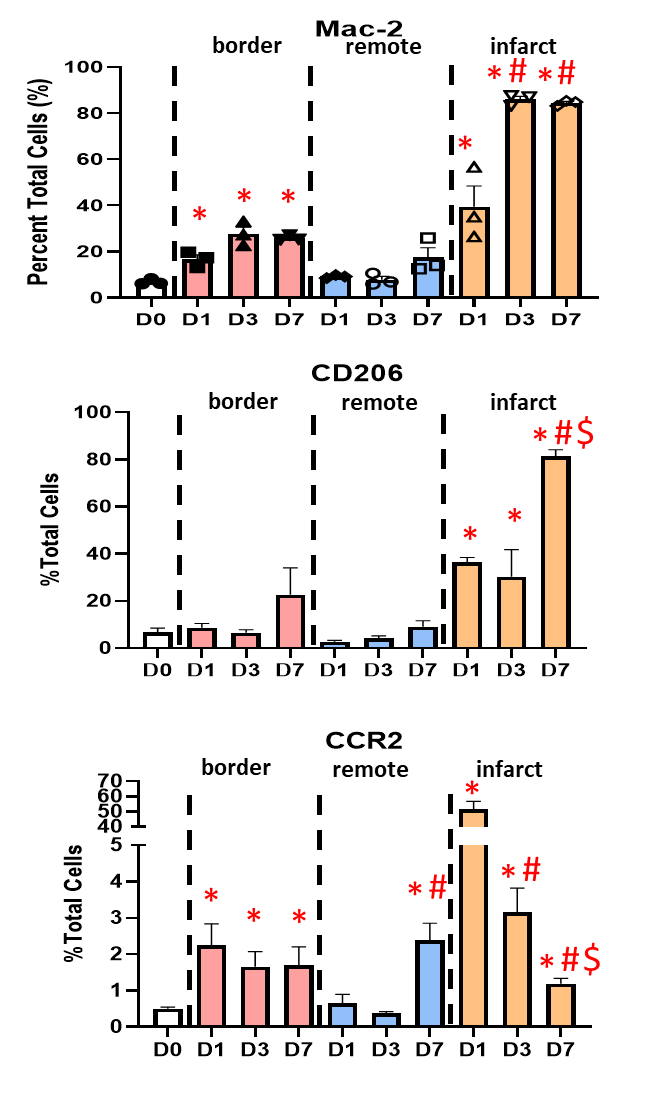
**

**Supplementary Figure 2.** Distribution of different macrophage subsets (total—Mac-2, M2—CD206, M1 or monocyte-derived—CCR2) in the infarcted heart by immunofluorescence, including border, infarct, and remote areas at days 1, 3, and 7 after MI. Primary antibody specificity was confirmed by staining with secondary antibody (2° Ab) only with the listed Opal fluorophores. *p<0.05 versus no MI, #p<0.05 versus D1, $p<0.05 versus D3.


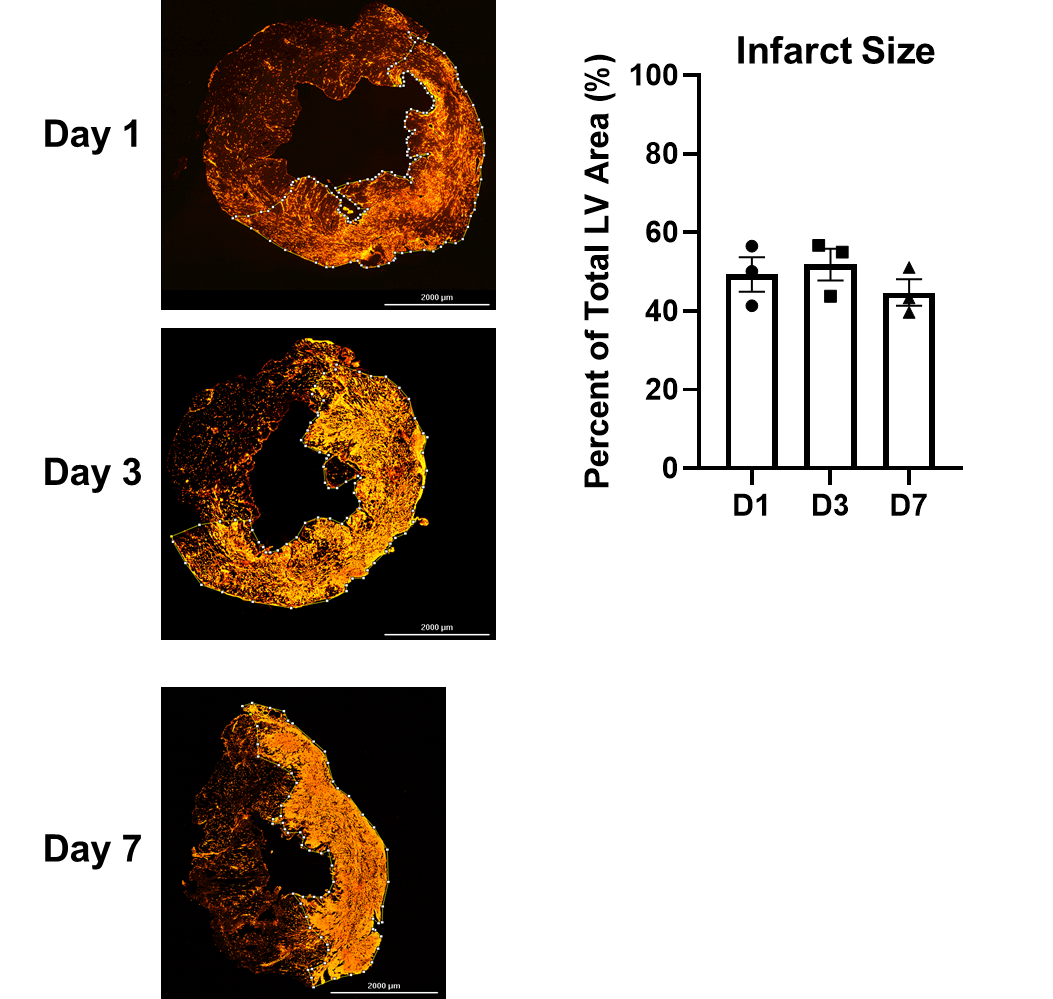


**Supplementary Figure 3.**

**Supplemental Figure 3.** Infarct size by Mac-2 staining at days 1, 3, and 7 post-MI.

**Supplementary Figure 4.**

**
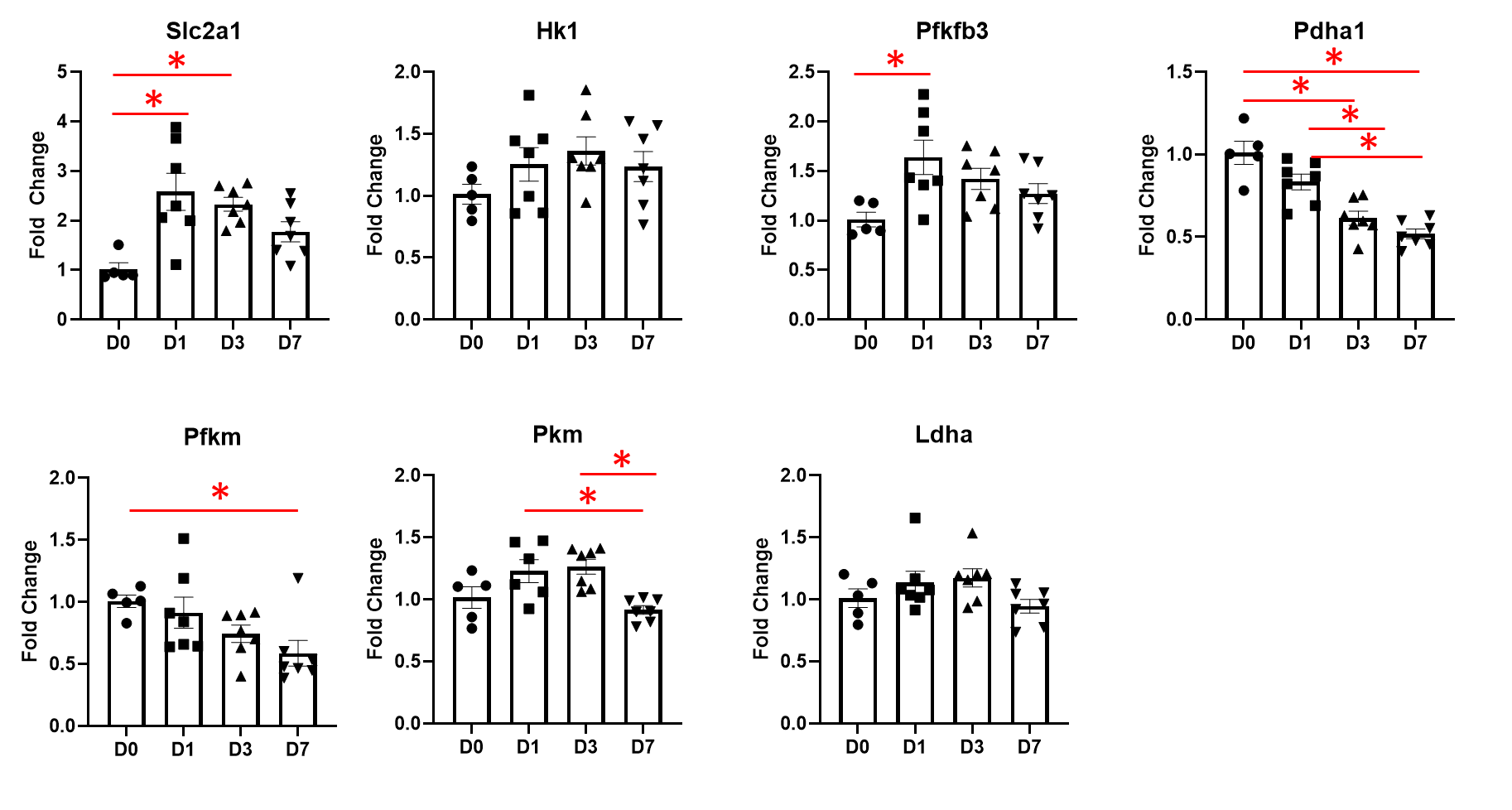
**

**Supplementary Figure 4.** Expression of glycolytic genes in the remote area of the heart. *p<0.05.

**Supplementary Figure 5.**


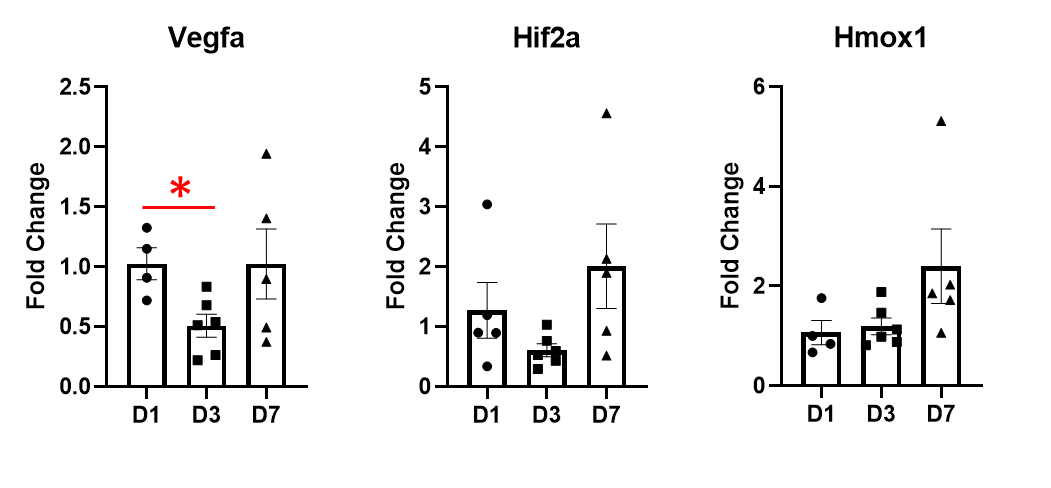


**Supplementary Figure 5.** Expression of Vegfa, Hif2a, and Hmox1 in infarct macrophages.

**Supplementary Figure 6.**


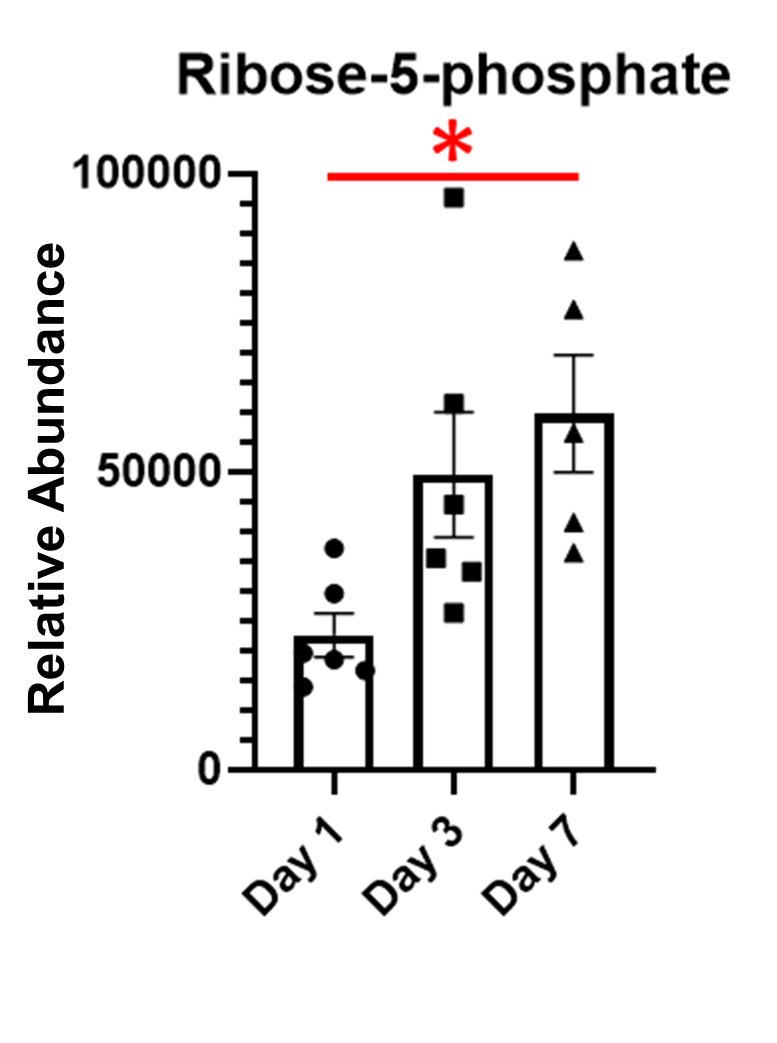


**Supplemental Figure 6.** Levels of ribose-5-phosphate as assessed by liquid chromatography-mass spectrometry in isolated infarct macrophages at days 1, 3, and 7 post-MI. *p<0.05.

**Supplementary Figure 7.**

**
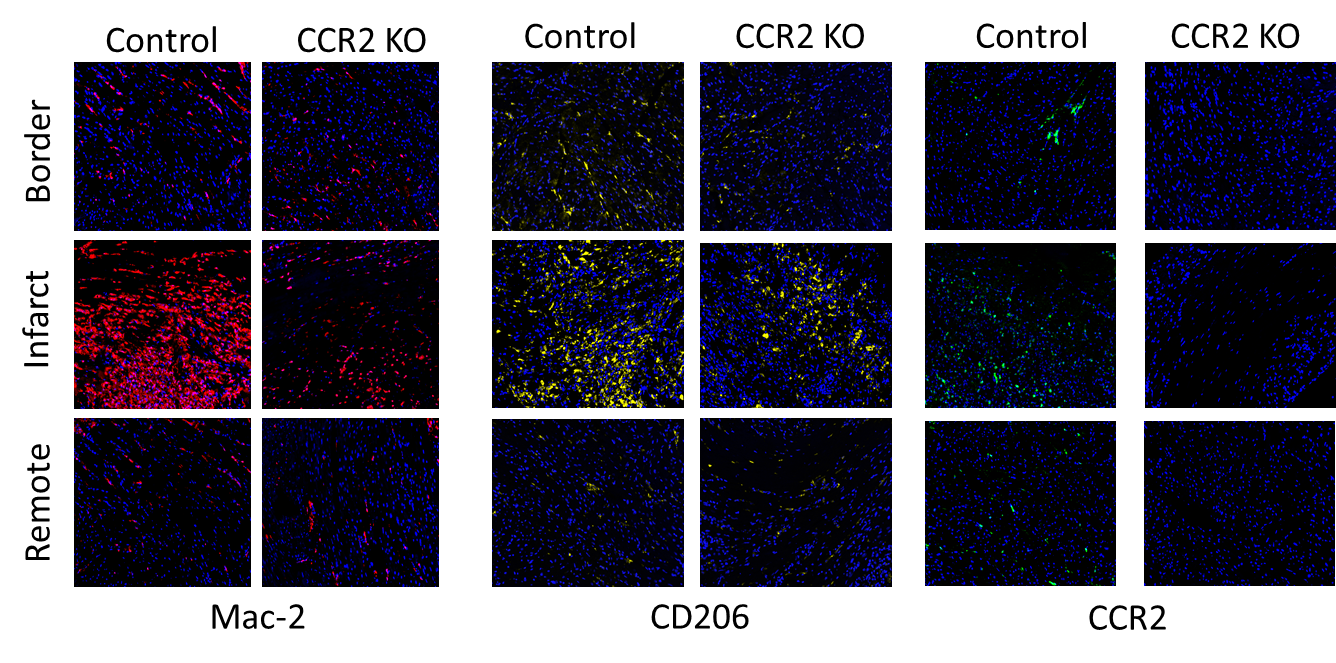
**


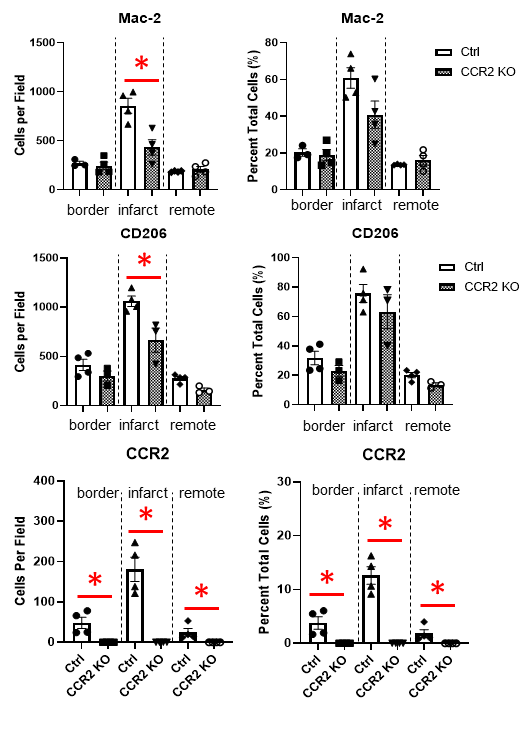


**Supplemental Figure 7.** Distribution of macrophage subsets by immunofluorescence in control C57BL/6J mice or CCR2 KO mice at day 3 post-MI. *p<0.05.

**Supplementary Figure 8.**

**
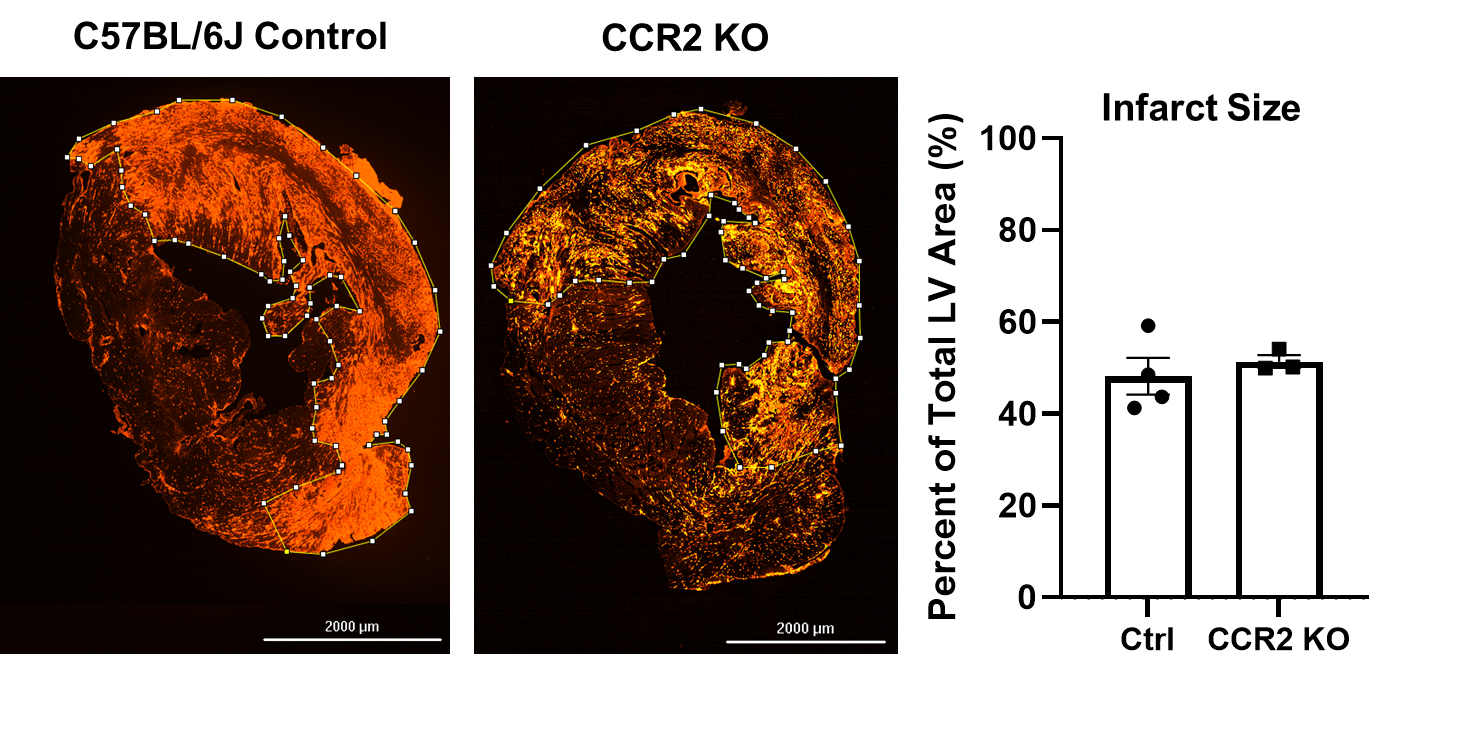
**

**Supplemental Figure 8.** Infarct size by Mac-2 staining at day 3 post-MI in control C57BL/6J mice and CCR2 KO mice.

**Supplementary Figure 9.**


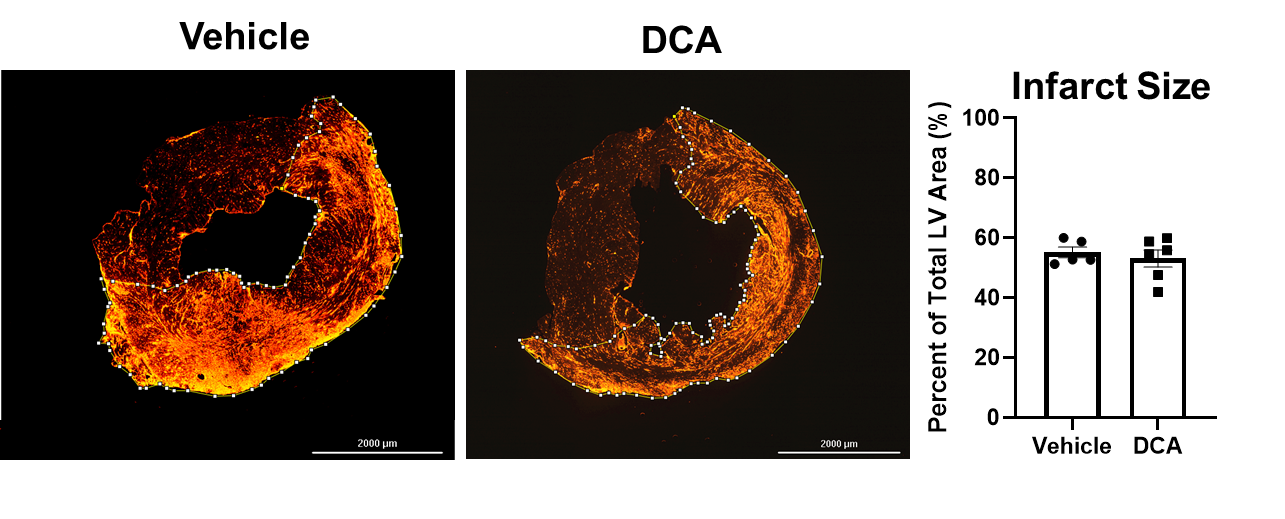


**Supplemental Figure 9.** Infarct size by Mac-2 staining at day 3 post-MI in vehicle or DCA-treated mice.

**Supplementary Figure 10.**

**
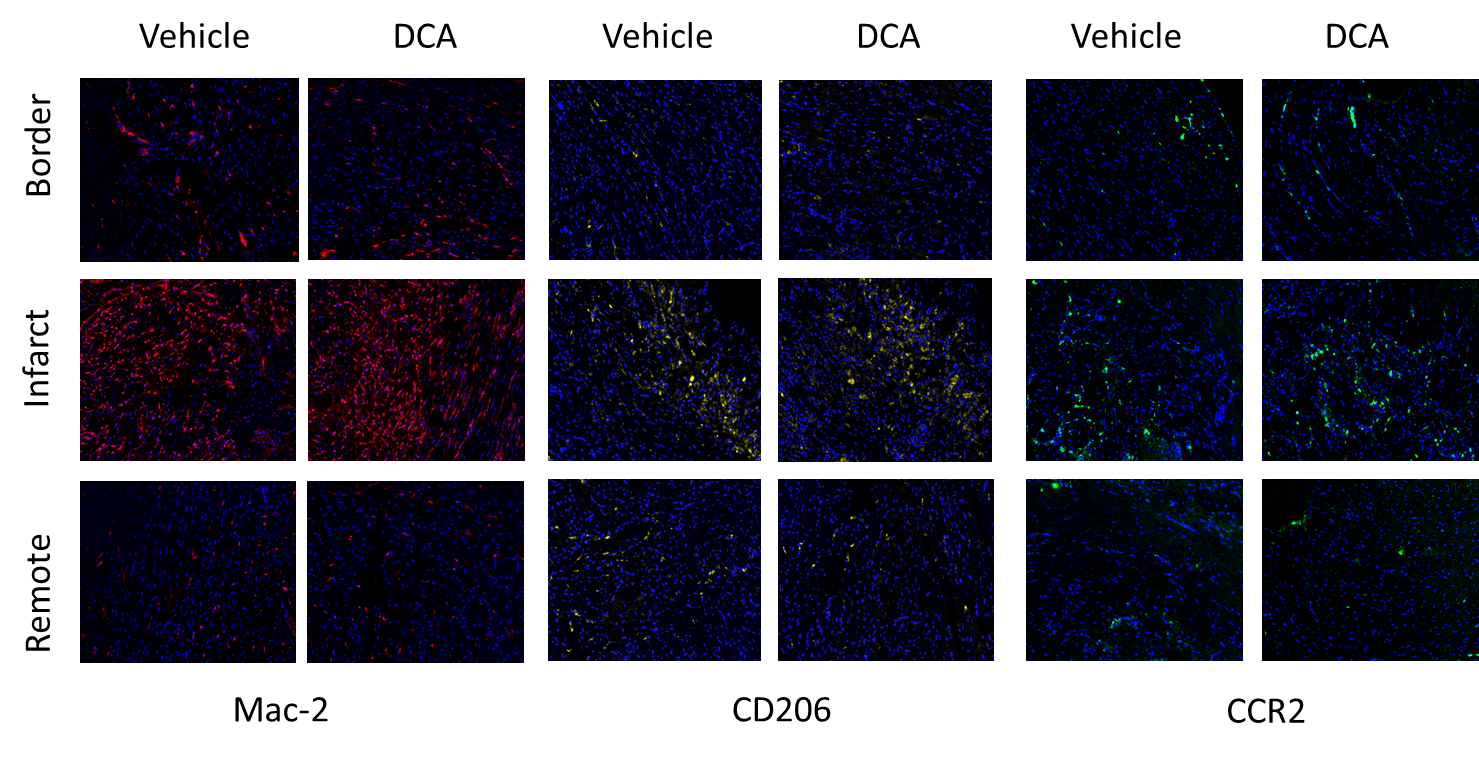
**


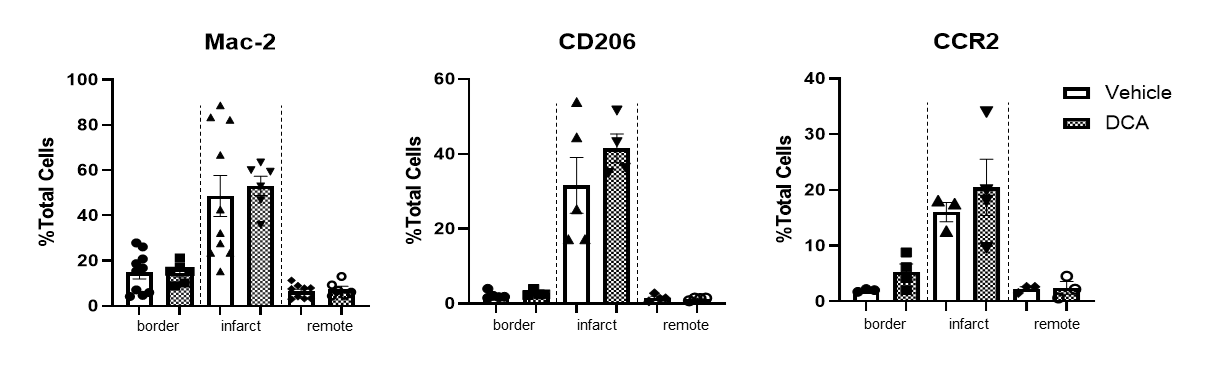


**Supplemental Figure 10.** Distribution of macrophage subsets by immunofluorescence in vehicle or DCA-treated mice at day 3 post-MI.
